# New approaches of N-acetylcysteine on fatty acid transport and metabolism in a rat model of MASLD induced by high-fat diet

**DOI:** 10.1038/s41598-026-55583-w

**Published:** 2026-05-31

**Authors:** Klaudia Sztolsztener, Brintha Croos, Adrian Chabowski

**Affiliations:** 1 Department of Physiology, Medical University of Bialystok, Bialystok, Poland; 2 College of Humanities and Sciences, Chemistry, Environmental Science, and Geology Department, University of Mary Hardin-Baylor, Texas, United States

**Keywords:** MASLD, n-acetylcysteine, Fatty acids transportation, Lipid metabolism, Fatty acids, High-fat diet, Liver diseases, Steatosis, Biochemistry, Diseases, Gastroenterology, Medical research, Physiology

## Abstract

The number of individuals suffering from metabolic dysfunction-associated steatotic liver disease (MASLD) has increased. The worldwide occurrence of fatty liver diseases is estimated to affect between 30% and 38% of adults from all races, ethnic group and sex in diet dependent manner, positioning these conditions as leading causes of chronic liver diseases. It is crucial to identify a natural substance that can safeguard against alterations in the lipid balance. There is a significant amount of research indicating that n-acetylcysteine (NAC) helps prevent inflammation and lipid deposition in peripheral tissues. The aim of this study was to investigate the effects of NAC on lipid metabolism and fatty acid composition in the liver of rats fed a high-fat diet (HFD). An experiment was conducted on male Wistar rats that received a standard diet or an HFD, divided into 4 groups (*n* = 6). Half of the rats from the Control and HFD groups received and intragastrically NAC solution. After 8 weeks of experimental procedures, rats were anaesthetized, and the liver tissue was used for further analysis. Gas-liquid chromatography was used to determine the content of total lipid fractions and fatty acid composition in each fraction. Western blot and real-time PCR methods were used to measure the expression of protein or mRNA of fatty acid (FA) transporters and enzymes that regulated lipid metabolism. NAC decreased FA transport into hepatocytes by a decline in the expression of FATP2, FABPpm, and CD36. Supplementation of NAC also significantly reduced elongation of C16:0, PA to C18:0 and enlarged the C20:0/C18:0 elongation ratio with simultaneous enhancement in C20:5 n-3, EPA and C22:6 n-3, DHA levels. Based on our results, we concluded that NAC point anti-inflammatory and pro-resolving properties, suggesting its potential role in the limitation of the development of simple steatosis changes by altering lipid disruption in rats receiving a high-fat diet.

**Supplementary Information:**

The online version contains supplementary material available at 10.1038/s41598-026-55583-w.

## Introduction

The number of patients with metabolic dysfunction-associated steatotic liver disease (MASLD) has increased in countries with a predominant Western diet^1^. Nowadays, MASLD prevalence ranges from 30% to 38% and affects people from all races, ethnic groups and sex in diet dependent manner, and its prevalence is increasing all over the world^1–3^. It is important to note that in the last years, the terminology of liver steatosis disorders has changed a few times. Currently, MASLD is defined as a liver disorder in which excess lipid deposits in the triacylglycerol (TAG) pool cover at least 5% of the hepatocyte cytoplasm space^4^. For the proper diagnosis of MASLD, at least one of three additional metabolic factors, including overweight or obesity, type 2 diabetes, and cardiometabolic risk criteria, must also be diagnosed^5^. Systemic metabolic disorders can disrupt lipid homeostasis and glucose metabolism, potentiating the hepatic accumulation of lipids that successively simple steatosis, worsening the systemic metabolic function^6^. Hyperlipidemia and hyperglycemia alter the activity of key enzymes that regulate lipid metabolism, promoting enhanced lipolysis in adipose tissue and accelerating the hepatic fatty acids (FA) influx and hepatic *de novo* lipogenesis (DNL)^7,8^. In hepatic steatosis lipid pool, namely FA, constitutes the major part, incorporating other lipid species, and is finally converted to TAG under esterification and stored in the form of lipid droplets. So, the mentioned alterations are considered the main metabolic defects related to MASLD development and progression. In other FA metabolism pathways, they can be oxidized into multiple forms, including structures with dissimilar numbers of double bonds, chain lengths, and desaturation degrees^6^. Additionally, lipid disposal capacities, like the β-oxidation of FA, are also impaired in the fatty liver condition. As hepatic steatosis develops, FAs are susceptible to being oxidized to saturated fatty acids (SFA) form, including palmitic acid (C16:0, PA) and stearic acid (C18:0, SA), which are then converted to monounsaturated fatty acids (MUFA) under stearoyl-CoA desaturase 1 (SCD1) activity^6^. On the other hand, recently, data reported that the hepatic composition of fatty acids is a feature determining insulin resistance and decreasing the activity of SCD1, which finally exacerbates hepatocellular disease and fibrosis^9^. Mentioned reports have suggested a relation between the content and composition of FA accumulated in the liver tissue and MASLD development and progression to steatohepatitis; thus, it is important to find a natural agent that limits changes in the composition ratios of FA and the expression or activity of enzymes regulating them. Extensive literature data describes the preventive role of n-acetylcysteine (NAC) on the inflammatory response and lipid accumulation in peripheral tissues under conditions of lipid and glucose overload^10–12^. NAC is a natural compound and due to the presence of thiol groups in its structure, it contains a free sulfhydryl group that reduces disulfide bonds, disclosing antioxidant effects. By increasing reduced glutathione (GSH) levels, NAC enhances the cellular capacity to neutralize reactive oxygen species (ROS). Excessive ROS generation in this context leads to lipid peroxidation and exacerbates liver damage. Therefore, interventions that enhance antioxidant defenses may attenuate these pathological processes^10^. By restoring redox balance and limiting ROS-mediated damage, NAC likely contributes to the attenuation of hepatic lipid accumulation and associated liver alterations observed in our model. A reduction in the n-6/n-3 PUFA ratio limits the production of pro-inflammatory eicosanoids while promoting the formation of inflammation-resolving mediators^13^. Consequently, this shift in fatty acid composition supports an anti-inflammatory effect and may contribute to the attenuation of inflammation associated with hepatic steatosis. Nevertheless, NAC shows beneficial effects on lipid and glucose metabolism through partially reducing the plasma levels of triglycerides, total cholesterol fractions in rats subjected to a high-fat diet^12^. Its administration in a short-term manner prevented weight gain and dyslipidemia induced by a high-sucrose diet, suggesting its potential applications in metabolic disturbances^10^. However, the available reports were not clarified and do not clearly demonstrate NAC influence. In the herein study, we examined how NAC could play a role in fatty liver disease, focusing on the composition and ratios of FA and lipotoxicity related to them. This study allowed for the assessment of NAC effects on the hepatic metabolism of fatty acids by measuring the expression of enzymes that regulate FA metabolic pathways. Moreover, we measured the expression of protein and gene of the fatty acid transporters and their changes under NAC treatment in the lipid overload condition. Additionally, the study sought to determine whether NAC could limit the development of hepatic steatosis by modulating fatty acid transport and metabolic pathways.

## Materials and methods

### Experimental procedures

All animal procedures were conducted on male Wistar rats initially weighing between 50 and 70 g. The decision to start with younger animals was made to avoid potential age-related confounding factors, e.g., to increase responsiveness to dietary intervention. Rats were maintained under standard holding conditions, which included unrestricted access to water and standard rodent chow, as well as controlled environmental parameters (22 ± 2 °C, 55 ± 10% humidity, and a reversed 12-hour light/dark cycle) for one week to acclimatize. Following the acclimatization period, animals were randomly assigned to experimental groups, with six rats allocated to each group: (1) Control – animals fed with a standard diet characterized by regular fat content, comprising an energy distribution of 65.5% carbohydrates, 24.2% proteins, and 10.3% fats; (2) HFD – animals fed with an high-fat diet (HFD) characterized by a high fat content with an energy distribution of 59.8% fats, 20.1% proteins, and 20.1% carbohydrates; (3) NAC – animals fed with a standard diet supplemented with N-acetylcysteine (NAC) (Sigma Aldrich, Saint Louis, MO, USA); 4) HFD + NAC – animals fed with an high-fat diet supplemented with N-acetylcysteine. Sample size was determined using open-access tools (http://biomath.info) and in accordance with 3R rules. Standard rodent diet was provided from Agropol company (Motycz, Poland), and experimental high-fat diet was obtained from Research Diet company (New Brunswick, NJ, USA). The nutritional composition of both diets was presented in our previous research^14^. The weight of the actual food was in line with the manufacturer’s recommendations and updated every two days based on the current body weight (body mass was previously published^15^. The concentration of N-acetylcysteine was set at a dosage of 500 mg/kg of body weight, which was carefully monitored every two days. Antioxidant solution was made by dissolving NAC powder in a saline solution (the same volume every day for all rats) and subsequently administered intragastrically once daily for the duration of an eight-week experiment. At the end of the experiment (8 weeks), animals were anesthetized. During the procedure for euthanasia, the animals were gently restrained and an appropriate size of needle was used; then, the calculated anesthetic dose of sodium pentobarbital was injected (at 80 mg/kg of final body mass). Following the injection, animals were euthanized by bleeding them to death (from the inferior vena cava). Liver tissue was then promptly excised, frozen using a temperature of liquid nitrogen, and stored at -80 °C for subsequent analyses. The current study continues our previous research using the same group of animals described earlier^14,15^. The liver tissue collected from that experiment was split for multiple analyses, each examining different mechanistic pathways relevant to MASLD development. The main reagents used in this study are presented in Table S1.

All procedures on animals were conducted under the approval of the Local Ethical Committee for Animal Experiments at the Medical University of Bialystok (Approval No. 21/2017).

### Gas-liquid chromatography assay

Liver tissue was used for the extraction of lipids using a chloroform and methanol solution (2:1, *v/v*) in accordance with the Folch method^16^. Resulted lipid extracts were added to the new tube containing internal standard, a mixture of heptadecanoic acid, diheptadecanoic acid, and triheptadecanoic acid, distributed on silica glass chromatography coated with gel (Silica Plate 60, 0.25 mm, Merck, Darmstadt, Germany). The separation into individual fractions, namely triacylglycerol (TAG), diacylglycerol (DAG), free fatty acid (FFA), and phospholipid (PL), was provided using thin-layer chromatography (TLC) by the addition of a special separation buffer (heptane/isopropyl ether/acetic acid at 60:40:3, *v/v/v*). Eluents with separated lipid fractions were transmethylated in a 14% boron trifluoride-methanol solution and dissolved in hexane solution. The particular fatty acid methyl esters (FAME) content in all lipid fractions was counted depending on the standards’ retention time using a gas-liquid chromatography (GLC; Hewlett-Packard 5890 Series II gas chromatograph) fitted into a Hewlett-Packard-INNOWax capillary column and flame ionization detector (Agilent Technologies, Santa Clara, CA, USA).

According to the composition of fatty acid the total content of fractions content of TAG, DAG, FFA, PL were estimated (C14:0, MA- myristic acid, C16:0, PA - palmitic acid, C16:1, PoA - palmitoleic acid, C18:0, SA - stearic acid, C18:1, OA - oleic acid, C18:2 n-6, LA - linoleic acid, C20:0 - arachidic acid, C18:3 n-3, ALA - linolenic acid, C22:0, BA - behenic acid, C20:4 n-6, AA - arachidonic acid, C24:0, LiA - lignoceric acid, C20:5 n-3, EPA - eicosapentaenoic acid, C24:1, NA - nervonic acid, C22:6 n-3, DHA - docosahexaenoic acid) and expressed in nanomoles per gram of tissue. The content of SFAs (as the sum of C14:0 MA, C16:0 PA, C18:0 SA, C20:0, C22:0 BA, and C24:0 LiA), MUFAs (as the sum of C16:1 PoA, C18:1 OA, and C24:1 NA), polyunsaturated fatty acid (PUFA; as the sum of C18:2 n-6 LA, C18:3 n-3 ALA, C20:4 n-6 AA, C20:5 n-3 EPA, and C22:6 n-3 DHA) in all examined lipid fractions was also assessed. The concentration of arachidonic acid was presented in our previous article^17^.

### Real-time quantitative polymerase chain reaction assay

Total RNA fraction was isolated from liver tissue using a TRIzol Reagent (Sigma Aldrich, Saint Louis, MO, USA) in accordance with the manufacturer’s protocol. Assessment of quantity and quality of RNA was performed as spectrophotometric absorbance O.D. (260/280 nm ratio) using a microplate Synergy H1 Hybrid Reader (BioTek Instruments, Winooski, VT, USA). Obtained RNA fraction in an amount of 1 µg was used to synthesize first-strand cDNA using an EvoScript Universal cDNA Master kit (Roche Molecular Systems, Boston, MA, USA), subjected to the following conditions: 42 °C for 15 min, 85 °C for 5 min, 65 °C for 15 min, and 4 °C for at least 4 min. After that quantitative real-time polymerase chain reaction (qRT-PCR) was carried out by the use of FastStart Essential DNA Green Master kit (Roche Molecular Systems, Boston, MA, USA) in accordance with the following conditions: preincubation, 3-step amplification for 45 s, 15 s for each step, (denaturation at 95 °C, annealing in the proper primers’ temperature, extension at 72 °C) and finally melting. Table 1 presents the details of the primers’ sequences and their annealing temperature. All reactions were conducted in duplicate on the LightCycler 96 Instrument with a real-time thermal cycler (Roche Diagnostics, Boston, MA, USA). Specificity of PCR products was confirmed based on a proper melting curve. Melt curve analysis revealed a single, sharp peak for each primer pair, indicating amplification of a single, specific product with no detectable primer dimers or non-specific products. Gene expression was normalized to housekeeping gene expression, as follows: glyceraldehyde-3-phosphate dehydrogenase (GAPDH), and quantified to the relative quantification method modified by Pfaffl^18^. Protein expression was normalized to total protein expression (Control group) and set as 100%. mRNA gene expression was set as 1.

**Table 1:** Primer sequences used for RT-PCR assay.

Target Gene	Forward Primer Sequences (5′→3′)	Reverse Primer Sequence (5′→3′)
*Cd36*	GCCTCCTTTCCACCTTTTGT	GATTCAAACACAGCATAGATGGAC
*Fabppm*	TCATCCTTTGTCTCCAGCTTTT	CCTATGCCATGCTGACAGGT
*Fatp2*	AGTACATCGGTGAACTGCTTCGGT	TGCCTTCAGTGGAAGCGTAGAACT
*Fatp5*	TTCAGGGACCACTGGACTTCCAAA	ACCACATCATCAGCTGTTCTCCCA
*Gapdh*	TGCACCACCAACTGCTTA	GGATGCAGGGATGATGTTC

*Cd36* – fatty acid translocase; *Fabppm* – membrane-associated fatty acid binding protein; *Fatp2* and *Fatp5* – fatty acid transporter protein 2 and 5; *Gapdh* – glyceraldehyde-3-phosphate dehydrogenase.

### Western blot assay

The liver samples were homogenized in ice-cold radioimmunoprecipitation assay (RIPA) buffer with the addition of protease and phosphatase inhibitors (Roche Diagnostics GmbH, Mannheim, Germany). Following the incubation (at 4 °C for 45 min), samples were centrifuged at 10,000 ×g 4 °C for 30 min, and in the resulting supernatant fraction, the protein concentration was assessed using a bicinchoninic acid (BCA) technique with bovine serum albumin (BSA) as a standard. Based on the protein concentration, the supernatant was restored to the same protein mass by the addition of Laemmli buffer (Bio-Rad, Hercules, CA, USA). Next, samples were loaded on the Criterion TGX Stain-Free Precast Gels (Bio-Rad, Hercules, CA, USA) and separated under electrophoresis. Following transfer onto polyvinylidene fluoride (PVDF) or nitrocellulose membranes for semi-dry or wet transfers, respectively, membranes were incubated with 5% blocking buffer prepared from tris-buffered saline with Tween 20 (TBST) with the addition of BSA or skimmed milk powder, and then were subjected to overnight immunoblotting with a proper primary antibody. Table S2 presents the details of primary antibodies and their dilutions. The second day, membranes were subjected to a proper secondary antibody conjugated with horseradish peroxidase (HRP) for the visualization of protein bands after the addition of Clarity Western ECL substrate (Bio-Rad, Hercules, CA, USA). The resulting protein band signal was densitometrically measured using the ChemiDoc visualization system fitted into Image Laboratory software (version 6.0.1, Bio-Rad, Warsaw, Poland). Protein expression was normalized to total protein expression (Control group), setting of 100% (normalization by sum using total protein staining). All samples were run at the same time point and gel to keep the constant conditions. After quantification and export to an image file, representative bands were cut and then presented on the graphs.

The images of whole gels showing the expression of selected proteins and the total protein loading in the liver tissue in the experimental groups are presented in the supplementary data (Figure S1 – S16).

### Statistical analysis

Statistical analyses were performed using GraphPad Prism version 10.2.3 (GraphPad Software, San Diego, CA, USA). Results are expressed as mean ± standard deviation (SD) from six independent determinations (*n* = 6). The normality of data distribution was assessed using the Shapiro-Wilk test. A two-way analysis of variance (ANOVA) was applied to evaluate the effects of high-fat diet (HFD) and N-acetylcysteine (NAC), as well as their interaction for the comparisons between HFD, NAC and HFD + NAC groups versus Control group. Results are reported as *F* and *p* values for each effect. Person correlation coefficients was used to measure linear correlation between fatty acid content. The statistical comparison between HFD + NAC and HFD groups was assessed by the parametric *t*-test or the non-parametric Mann-Whitney U test. A difference was considered statistically significant at *p* < 0.05.

## Results

### Impact of n-acetylcysteine on the hepatic lipid content

In triacylglycerol (TAG) fraction saturated fatty acid (SFA), monounsaturated fatty acid (MUFA) and polyunsaturated fatty acid (PUFA) levels was changed (SFA – HFD effect: F(1,13) = 76.47, *p* < 0.0001, NAC effect: F(1,13) = 0.7582, *p* = 0.3997, interaction: F(1,13) = 0.006259, *p* = 0.9381; MUFA – HFD effect: F(1,14) = 137.9, *p* < 0.0001, NAC effect: F(1,14) = 0.5603, *p* = 0.4665, interaction: F(1,14) = 0.2038, *p* = 0.6586; PUFA – HFD effect: F(1,13) = 58.67, *p* < 0.0001, NAC effect: F(1,13) = 1.776, *p* = 0.2055, interaction: F(1,13) = 0.1188, *p* = 0.7358; vs. Control group, Table 2). Overall, total TAG content was also different in rats from all experimental groups (HFD effect: F(1,14) = 69.56, *p* < 0.0001, NAC effect: F(1,14) = 2.575, *p* = 0.1308, interaction: F(1,14) = 1.035, *p* = 0.3263; vs. Control group, Table 2).

In diacylglycerol (DAG) fraction the administration of HFD or/with NAC alternated SFA, MUFA and PUFA ratios (SFA – HFD effect: F(1,13) = 6.554, *p* = 0.0237, NAC effect: F(1,13) = 8.462, *p* = 0.0122, interaction: F(1,13) = 0.9253, *p* = 0.3536; MUFA – HFD effect: F(1,21) = 11.28, *p* = 0.0030, NAC effect: F(1,21) = 0.02489, *p* = 0.8761, interaction: F(1,21) = 3.334, *p* = 0.0821; PUFA – HFD effect: F(1,21) = 0.1692, *p* = 0.6850, NAC effect: F(1,21) = 5.482, *p* = 0.0292, interaction: F(1,21) = 0.02853, *p* = 0.8675; vs. Control group, Table 2). In all examined groups total DAG was changed (HFD effect: F(1,16) = 1.009, *p* = 0.3300, NAC effect: F(1,16) = 3.959, *p* = 0.0640, interaction: F(1,16) = 0.5359, *p* = 0.4747; vs. Control group, Table 2).

In free fatty acid (FFA) fraction, we observed an alteration in SFA, MUFA and PUFA ratios (SFA – HFD effect: F(1,13) = 31.27, *p* < 0.0001, NAC effect: F(1,13) = 0.002326, *p* = 0.9623, interaction: F(1,13) = 0.1805, *p* = 0.6779; MUFA – HFD effect: F(1,16) = 6.635, *p* = 0.0203, NAC effect: F(1,16) = 2.561, *p* = 0.1291, interaction: F(1,16) = 8.511, *p* = 0.0101; PUFA – HFD effect: F(1,20) = 0.6275, *p* = 0.4376, NAC effect: F(1,20) = 6.039, *p* = 0.0232, interaction: F(1,20) = 6.475, *p* = 0.0193; vs. Control group, Table 2). Overall, total FFA content was also different in rats from all experimental groups (HFD effect: F(1,24) = 0.8333, *p* = 0.3704, NAC effect: F(1,24) = 4.951, *p* = 0.0357, interaction: F(1,24) = 4.310, *p* = 0.0488; vs. Control group, Table 2).

In phospholipid (PL) fraction raised SFA, MUFA, PUFA levels was observed (SFA – HFD effect: F(1,30) = 248.4, *p* < 0.0001, NAC effect: F(1,30) = 0.06635, *p* = 0.7985, interaction: F(1,30) = 2.229, *p* = 0.1459; MUFA – HFD effect: F(1,29) = 82.82, *p* < 0.0001, NAC effect: F(1,29) = 1.008, *p* = 0.3237, interaction: F(1,29) = 1.947, *p* = 0.1735; PUFA – HFD effect: F(1,26) = 183.0, *p* < 0.0001, NAC effect: F(1,26) = 0.8540, *p* = 0.3639, interaction: F(1,26) = 7.301, *p* = 0.0120; vs. Control group, Table 2). The entire PL pool level was also changed under experimental condition (HFD effect: F(1,34) = 114.3, *p* < 0.0001, NAC effect: F(1,34) = 0.1562, *p* = 0.6952, interaction: F(1,34) = 0.04761, *p* = 0.8286; vs. Control group, Table 2). The concentration of PUFA in PL pool was lowered after NAC treatment of rats fed with an HFD (HFD + NAC: -7.0%, vs. HFD group, *p* < 0.05, Table 2).

**Table 2:** N-acetylcysteine (NAC) influence on the saturated (SFA), monounsaturated (MUFA), and polyunsaturated (PUFA) fatty acids in triacylglycerol (TAG), diacylglycerol (DAG), free fatty acid (FFA), phospholipid (PL) and total lipid fractions in the liver tissue of rats subjected to a standard diet (Control) or a high-fat diet (HFD). The values obtained in gas-liquid chromatography (GLC) method are expressed in nanomoles per gram of tissue and presented as mean ± standard deviation (SD); *n* = 6 rats per group. Statistical analysis was performed using two-way ANOVA, *t*-test or Mann-Whitney U test. A difference was considered statistically significant at *p* < 0.05 and marked as ^*^ - for the comparisons between HFD, NAC and HFD + NAC groups versus Control group and as ^#^ - for the comparison between HFD + NAC group versus HFD group.

		**Control**	**HFD**	**NAC**	**HFD + NAC**
TAG	SFA	14412.48 ± 1963.13	52412.49 ± 10595.09 ^*^	11003.26 ± 2017.66	48321.89 ± 11024.34
	MUFA	6783.63 ± 1551.05	45761.19 ± 9946.94 ^*^	5834.03 ± 1484.05	41925.93 ± 7917.55
	PUFA	17694.25 ± 3405.14	59087.59 ± 12849.98 ^*^	12584.23 ± 2003.93	50412.42 ± 13057.38
	Total TAG	37718.67 ± 7741.05	189115.84 ± 52154.28 ^*^	28210.28 ± 5055.31	146692.68 ± 33232.67
DAG	SFA	820.59 ± 30.79	880.26 ± 111.62 ^*^	676.06 ± 32.61 ^*^	807.56 ± 91.15
	MUFA	314.96 ± 58.53	367.22 ± 59.69 ^*^	247.33 ± 56.88	424.09 ± 104.68
	PUFA	866.45 ± 31.32	839.57 ± 123.56	750.15 ± 98.84 ^*^	738.92 ± 143.76
	Total DAG	2005.32 ± 58.51	2037.86 ± 274.91	1680.48 ± 326.95	1887.76 ± 263.62
FFA	SFA	676.88 ± 110.83	496.64 ± 35.97 ^*^	690.01 ± 76.68	480.14 ± 58.60
	MUFA	253.28 ± 51.81	245.20 ± 26.11 ^*^	146.48 ± 36.60	276.34 ± 61.83 ^*^
	PUFA	687.39 ± 133.96	543.04 ± 35.64	471.01 ± 24.35 ^*^	546.81 ± 139.69 ^*^
	Total FFA	1568.61 ± 258.38	1275.07 ± 77.37	1146.19 ± 89.48 ^*^	1260.43 ± 351.17 ^*^
PL	SFA	42840.50 ± 3391.00	66251.07 ± 5525.53	44516.75 ± 4187.76	63875.86 ± 2668.13
	MUFA	3325.14 ± 316.17	4657.85 ± 344.37 ^*^	3374.82 ± 330.73	4353.19 ± 425.84
	PUFA	49785.27 ± 4171.76	72729.57 ± 3465.08 ^*^	52299.01 ± 5386.18	67602.86 ± 2578.54 ^* #^
	Total PL	102388.74 ± 16128.91	141618.55 ± 11024.65 ^*^	101752.23 ± 10289.96	139412.70 ± 7069.06

SFA - saturated fatty acid; MUFA - monounsaturated fatty acid; PUFA - polyunsaturated fatty acid; TAG - triacylglycerol; DAG - diacylglycerol; FFA - free fatty acid; PL - phospholipid; HFD – high-fat diet; NAC - n-acetylcysteine.

Overall, HFD significantly altered the fatty acid subclasses composition and total content across TAG, DAG, FFA, and PL fractions, with consistent and strong diet effects observed for most lipid classes. NAC showed limited and fraction-specific effects, with few significant interactions, indicating that its impact on lipid remodeling was modest compared to the dominant effect of HFD.

### Impact of n-acetylcysteine on the hepatic fatty acids composition in triacylglycerol, diacylglycerol, phospholipid and free fatty acid fractions

**Table 3:** N-acetylcysteine (NAC) influence on the fatty acids composition in triacylglycerol (TAG) fraction in the liver tissue of rats subjected to a standard diet (Control) or a high-fat diet (HFD). The values are expressed in nanomoles per gram of tissue. The values obtained in gas-liquid chromatography (GLC) method are expressed in nanomoles per gram of tissue and presented as mean ± standard deviation (SD); *n* = 6 rats per group. Statistical analysis was performed using two-way ANOVA, *t*-test or Mann-Whitney U test. A difference was considered statistically significant at *p* < 0.05 and marked as ^*^ - for the comparisons between HFD, NAC and HFD + NAC groups versus Control group and as ^#^ - for the comparison between HFD + NAC group versus HFD group.

		**Control**	**HFD**	**NAC**	**HFD + NAC**
SFA	C14:0, MA	216.22 ± 42.92	1410.89 ± 348.71 ^*^	190.38 ± 49.99 ^*^	886.85 ± 228.35 ^* #^
	C16:0, PA	12222.16 ± 2779.68	45205.39 ± 9827.90 ^*^	9992.38 ± 1882.82	43659.78 ± 9914.71
	C18:0, SA	627.87 ± 85.10	5811.79 ± 1624.83 ^*^	724.17 ± 71.18	4613.37 ± 919.07 ^*^
	C20:0	31.53 ± 3.57	36.46 ± 8.52 ^*^	31.13 ± 5.76 ^*^	67.32 ± 8.76 ^* #^
	C22:0, BA	n/d	n/d	n/d	n/d
	C24:0, LiA	102.26 ± 18.40	114.15 ± 37.99 ^*^	44.34 ± 9.66 ^*^	97.20 ± 9.23 ^*^
MUFA	C16:1, PoA	808.06 ± 131.73	1075.66 ± 189.60 ^*^	922.86 ± 123.16	1100.09 ± 207.50
	C18:1, OA	6064.35 ± 1388.81	44685.53 ± 9782.90 ^*^	5034.04 ± 1242.86	40802.49 ± 7630.54
	C24:1, NA	n/d	n/d	n/d	n/d
PUFA	C18:2 n-6, LA	14364.99 ± 2855.23	46647.33 ± 10500.97 ^*^	9739.27 ± 1551.65	52008.81 ± 8363.23
	C18:3 n-3, ALA	1130.55 ± 290.91	3490.22 ± 941.55 ^*^	753.02 ± 203.15 ^*^	2696.65 ± 617.10
	C20:5 n-3, EPA	409.06 ± 5.01	491.59 ± 59.88 ^*^	427.87 ± 124.47 ^*^	622.11 ± 81.27 ^* #^
	C22:6 n-3, DHA	541.67 ± 48.92	2690.63 ± 667.13 ^*^	915.36 ± 212.59 ^*^	3469.25 ± 720.95

SFA - saturated fatty acid; MUFA - monounsaturated fatty acid; PUFA - polyunsaturated fatty acid; HFD – high-fat diet; NAC - n-acetylcysteine. n/d - not detected/below detection level.

In TAG fraction among SFA pool all examined fatty acid levels were changed (C14:0, MA – HFD effect: F(1,20) = 114.3, *p* < 0.0001, NAC effect: F(1,20) = 9.659, *p* = 0.0055, interaction: F(1,20) = 7.929, *p* = 0.0107; C16:0, PA – HFD effect: F(1,27) = 187.2, *p* < 0.0001, NAC effect: F(1,27) = 0.6008, *p* = 0.4450, interaction: F(1,27) = 0.01973, *p* = 0.8893; C18:0, SA – HFD effect: F(1,29) = 230.7, *p* < 0.0001, NAC effect: F(1,29) = 3.404, *p* = 0.0753, interaction: F(1,29) = 4.698, *p* = 0.0386; C20:0 – HFD effect: F(1,22) = 53.47, *p* < 0.0001, NAC effect: F(1,22) = 29.33, *p* < 0.0001, interaction: F(1,22) = 30.89, *p* < 0.0001; C24:0, LiA – HFD effect: F(1,21) = 17.09, *p* = 0.0005, NAC effect: F(1,21) = 22.86, *p* = 0.0001, interaction: F(1,21) = 6.841, *p* = 0.0162; vs. Control group, Table 3). In TAG fraction FA included in MUFA and PUFA pools were also altered under experimental conditions (C16:1, PoA – HFD effect: F(1,22) = 12.41, *p* = 0.0019, NAC effect: F(1,22) = 1.216, *p* = 0.2820, interaction: F(1,22) = 0.5123, *p* = 0.4817; C18:1, OA – HFD effect: F(1,26) = 299.7, *p* < 0.0001, NAC effect: F(1,26) = 1.308, *p* = 0.2633, interaction: F(1,26) = 0.4408, *p* = 0.5126; C18:2 n-6, LA – HFD effect: F(1,24) = 213.7, *p* < 0.0001, NAC effect: F(1,24) = 0.02082, *p* = 0.8865, interaction: F(1,24) = 3.836, *p* = 0.0619; C18:3 n-3, ALA – HFD effect: F(1,25) = 99.97, *p* < 0.0001, NAC effect: F(1,25) = 7.404, *p* = 0.0117, interaction: F(1,25) = 0.9344, *p* = 0.3430; C20:5 n-3, EPA – HFD effect: F(1,31) = 30.76, *p* < 0.0001, NAC effect: F(1,31) = 8.956, *p* = 0.0054, interaction: F(1,31) = 5.012, *p* = 0.0325; C22:6 n-3, DHA – HFD effect: F(1,26) = 191.1, *p* < 0.0001, NAC effect: F(1,26) = 11.47, *p* = 0.0023, interaction: F(1,26) = 1.417, *p* = 0.2447; vs. Control group, Table 3). Moreover, in comparison with the HFD group, in TAG fraction NAC administration to rats subjected to an HFD reduced the level of C14:0, MA (HFD + NAC: -37.1%, *p* < 0.05, Table 3) and enhanced C20:0 and C20:5 n-3, EPA levels (HFD + NAC: +84.6% and + 26.5%, respectively, *p* < 0.05, Table 3).

**Table 4:** N-acetylcysteine (NAC) influence on the fatty acids composition in diacylglycerol (DAG) fraction in the liver tissue of rats subjected to a standard diet (Control) or a high-fat diet (HFD). The values obtained in gas-liquid chromatography (GLC) method are expressed in nanomoles per gram of tissue and presented as mean ± standard deviation (SD); *n* = 6 rats per group. Statistical analysis was performed using two-way ANOVA, *t*-test or Mann-Whitney U test. A difference was considered statistically significant at *p* < 0.05 and marked as ^*^ - for the comparisons between HFD, NAC and HFD + NAC groups versus Control group and as ^#^ - for the comparison between HFD + NAC group versus HFD group.

		**Control**	**HFD**	**NAC**	**HFD + NAC**
SFA	C14:0, MA	62.55 ± 4.55	58.78 ± 4.87 ^*^	53.41 ± 8.15	78.79 ± 11.45 ^* #^
	C16:0, PA	564.14 ± 37.22	540.50 ± 59.39	436.35 ± 18.55 ^*^	519.40 ± 51.62 ^*^
	C18:0, SA	162.69 ± 12.30	233.73 ± 51.25 ^*^	163.13 ± 10.36	204.78 ± 27.97
	C20:0	8.42 ± 1.36	5.28 ± 1.33 ^*^	9.15 ± 0.91 ^*^	9.81 ± 2.20 ^* #^
	C22:0, BA	5.14 ± 1.10	4.32 ± 0.47	5.27 ± 0.33 ^*^	6.23 ± 1.79
	C24:0, LiA	6.19 ± 1.07	5.15 ± 0.69	6.71 ± 1.55 ^*^	6.45 ± 1.57
MUFA	C16:1, PoA	31.87 ± 7.04	15.77 ± 2.29 ^*^	36.34 ± 12.42	16.61 ± 1.70
	C18:1, OA	267.23 ± 46.30	347.34 ± 58.16 ^*^	204.41 ± 45.99	346.67 ± 51.97
	C24:1, NA	5.07 ± 1.22	4.11 ± 0.86 ^*^	3.12 ± 1.07	5.88 ± 0.97 ^* #^
PUFA	C18:2 n-6, LA	623.55 ± 52.89	474.61 ± 64.04 ^*^	441.77 ± 36.36 ^*^	466.90 ± 100.31 ^*^
	C18:3 n-3, ALA	35.58 ± 1.17	23.86 ± 2.45 ^*^	34.84 ± 5.75	31.60 ± 8.20 ^*^
	C20:5 n-3, EPA	8.99 ± 0.56	7.44 ± 0.72 ^*^	11.74 ± 3.68 ^*^	9.15 ± 1.72
	C22:6 n-3, DHA	43.69 ± 3.92	52.54 ± 6.22 ^*^	43.31 ± 9.11	53.30 ± 9.73

SFA - saturated fatty acid; MUFA - monounsaturated fatty acid; PUFA - polyunsaturated fatty acid; HFD – high-fat diet; NAC - n-acetylcysteine.

In DAG fraction fatty acid levels from SFA pools were changed (C14:0, MA – HFD effect: F(1,25) = 12.62, *p* = 0.0015, NAC effect: F(1,25) = 3.197, *p* = 0.0859, interaction: F(1,25) = 22.96, *p* < 0.0001; C16:0, PA – HFD effect: F(1,27) = 3.872, *p* = 0.0595, NAC effect: F(1,27) = 24.32, *p* < 0.0001, interaction: F(1,27) = 12.49, *p* = 0.0015; C18:0, SA – HFD effect: F(1,27) = 30.08, *p* < 0.0001, NAC effect: F(1,27) = 1.926, *p* = 0.1765, interaction: F(1,27) = 2.047, *p* = 0.1640; C20:0 – HFD effect: F(1,27) = 4.979, *p* = 0.0342, NAC effect: F(1,27) = 22.27, *p* < 0.0001, interaction: F(1,27) = 11.57, *p* = 0.0021; C22:0, BA – HFD effect: F(1,25) = 0.02480, *p* = 0.8761, NAC effect: F(1,25) = 5.352, *p* = 0.0292, interaction: F(1,25) = 4.044, *p* = 0.0552; C24:0, LiA – HFD effect: F(1,28) = 2.557, *p* = 0.1210, NAC effect: F(1,28) = 4.990, *p* = 0.0337, interaction: F(1,28) = 0.9294, *p* = 0.3433; vs. Control group, Table 4). In DAG fraction FA from MUFA and PUFA pools were also altered under experimental conditions (C16:1, PoA – HFD effect: F(1,28) = 45.28, *p* < 0.0001, NAC effect: F(1,28) = 0.9944, *p* = 0.3272, interaction: F(1,28) = 0.4614, *p* = 0.5026; C18:1, OA – HFD effect: F(1,33) = 47.65, *p* < 0.0001, NAC effect: F(1,33) = 3.884, *p* = 0.0572, interaction: F(1,33) = 3.722, *p* = 0.0623; C24:1, NA – HFD effect: F(1,28) = 6.200, *p* = 0.0190, NAC effect: F(1,28) = 0.05811, *p* = 0.8113, interaction: F(1,28) = 26.62, *p* < 0.0001; C18:2 n-6, LA – HFD effect: F(1,27) = 6.167, *p* = 0.0195, NAC effect: F(1,27) = 14.44, *p* = 0.0007, interaction: F(1,27) = 12.19, *p* = 0.0017; C18:3 n-3, ALA – HFD effect: F(1,31) = 19.00, *p* = 0.0001, NAC effect: F(1,31) = 4.148, *p* = 0.0503, interaction: F(1,31) = 6.096, *p* = 0.0193; C20:5 n-3, EPA – HFD effect: F(1,25) = 9.137, *p* = 0.0057, NAC effect: F(1,25) = 10.66, *p* = 0.0032, interaction: F(1,25) = 0.5822, *p* = 0.4526; C22:6 n-3, DHA – HFD effect: F(1,27) = 13.30, *p* = 0.0011, NAC effect: F(1,27) = 0.005743, *p* = 0.9402, interaction: F(1,27) = 0.04837, *p* = 0.8276; vs. Control group, Table 4). NAC supplementation to rats received an HFD provoked an increase in the DAG’s concentration of C14:0, MA, C20:0, C24:1, NA, C18:3 n-3, ALA (HFD + NAC: +34.0%, + 85.9%, + 43.1%, + 32.4%, respectively, *p* < 0.05, Table 4) compared to the HFD group.

**Table 5:** N-acetylcysteine (NAC) influence on the fatty acids composition in free fatty acid (FFA) fraction in the liver tissue of rats subjected to a standard diet (Control) or a high-fat diet (HFD). The values obtained in gas-liquid chromatography (GLC) method are expressed in nanomoles per gram of tissue and presented as mean ± standard deviation (SD); *n* = 6 rats per group. Statistical analysis was performed using two-way ANOVA, *t*-test or Mann-Whitney U test. A difference was considered statistically significant at *p* < 0.05 and marked as ^*^ - for the comparisons between HFD, NAC and HFD + NAC groups versus Control group and as ^#^ - for the comparison between HFD + NAC group versus HFD group.

		**Control**	**HFD**	**NAC**	**HFD + NAC**
SFA	C14:0, MA	34.78 ± 6.78	40.53 ± 6.49	28.61 ± 3.40 ^*^	34.47 ± 6.89 ^*^
	C16:0, PA	521.70 ± 101.30	322.53 ± 18.34 ^*^	383.17 ± 53.43 ^*^	305.97 ± 37.89 ^*^
	C18:0, SA	106.43 ± 11.33	139.51 ± 12.90 ^*^	105.26 ± 13.26	131.78 ± 25.69
	C20:0	5.94 ± 1.43	4.13 ± 0.54	4.42 ± 1.31	5.44 ± 1.01 ^*^
	C22:0, BA	2.47 ± 0.61	2.26 ± 0.39	1.93 ± 0.39	2.68 ± 0.48 ^*^
	C24:0, LiA	5.58 ± 1.47	3.96 ± 0.12 ^*^	166.70 ± 18.65 ^*^	3.18 ± 0.74 ^*^
MUFA	C16:1, PoA	45.89 ± 6.49	18.03 ± 1.90 ^*^	29.14 ± 6.24 ^*^	11.04 ± 1.88 ^* #^
	C18:1, OA	152.00 ± 23.24	217.20 ± 30.85 ^*^	107.31 ± 20.61 ^*^	194.27 ± 30.06
	C24:1, NA	2.97 ± 0.28	2.30 ± 0.16	2.49 ± 0.54	3.15 ± 0.62 ^*^
PUFA	C18:2 n-6, LA	389.65 ± 74.70	281.27 ± 15.96 ^*^	249.29 ± 23.14 ^*^	210.88 ± 65.85
	C18:3 n-3, ALA	47.33 ± 10.61	20.56 ± 2.61 ^*^	22.89 ± 4.34 ^*^	27.72 ± 6.14 ^* #^
	C20:5 n-3, EPA	15.03 ± 4.86	6.71 ± 1.24 ^*^	14.78 ± 0.35	7.81 ± 1.66
	C22:6 n-3, DHA	38.60 ± 3.31	26.67 ± 5.69	20.92 ± 3.56 ^*^	29.08 ± 4.47 ^*^

SFA - saturated fatty acid; MUFA - monounsaturated fatty acid; PUFA - polyunsaturated fatty acid; HFD – high-fat diet; NAC - n-acetylcysteine.

In FFA fraction experimental conditions increased or decreased level of FA from SFA group (C14:0, MA – HFD effect: F(1,26) = 7.749, *p* = 0.9785, NAC effect: F(1,26) = 8.606, *p* = 0.0069, interaction: F(1,26) = 0.0007422, *p* = 0.0099; C16:0, PA – HFD effect: F(1,22) = 40.31, *p* < 0.0001, NAC effect: F(1,22) = 12.69, *p* = 0.0017, interaction: F(1,22) = 7.851, *p* = 0.0104; C18:0, SA – HFD effect: F(1,27) = 27.31, *p* < 0.0001, NAC effect: F(1,27) = 0.6083, *p* = 0.4422, interaction: F(1,27) = 0.3312, *p* = 0.5697; C20:0 – HFD effect: F(1,28) = 1.162, *p* = 0.2902, NAC effect: F(1,28) = 0.08554, *p* = 0.7721, interaction: F(1,28) = 14.88, *p* = 0.0006; C22:0, BA – HFD effect: F(1,22) = 2.435, *p* = 0.1330, NAC effect: F(1,22) = 0.1131, *p* = 0.7398, interaction: F(1,22) = 7.796, *p* = 0.0106; C24:0, LiA – HFD effect: F(1,27) = 675.7, *p* < 0.0001, NAC effect: F(1,27) = 637.0, *p* < 0.0001, interaction: F(1,27) = 649.5, *p* < 0.0001; vs. Control group, Table 5). FFA’s FA from MUFA and PUFA pools were also changed (C16:1, PoA – HFD effect: F(1,18) = 186.3, *p* < 0.0001, NAC effect: F(1,18) = 49.66, *p* < 0.0001, interaction: F(1,18) = 8.405, *p* = 0.0096; C18:1, OA – HFD effect: F(1,26) = 64.02, *p* < 0.0001, NAC effect: F(1,26) = 12.65, *p* = 0.0015, interaction: F(1,26) = 1.310, *p* = 0.2629; C24:1, NA – HFD effect: F(1,21) = 0.005942, *p* = 0.9393, NAC effect: F(1,21) = 1.165, *p* = 0.2927, interaction: F(1,21) = 15.29, *p* = 0.0008; C18:2 n-6, LA – HFD effect: F(1,24) = 18.19, *p* = 0.0003, NAC effect: F(1,24) = 37.49, *p* < 0.0001, interaction: F(1,24) = 4.132, *p* = 0.0533; C18:3 n-3, ALA – HFD effect: F(1,30) = 31.96, *p* < 0.0001, NAC effect: F(1,30) = 19.85, *p* = 0.0001, interaction: F(1,30) = 66.35, *p* < 0.0001; C20:5 n-3, EPA – HFD effect: F(1,24) = 77.28, *p* < 0.0001, NAC effect: F(1,24) = 0.2420, *p* = 0.6272, interaction: F(1,24) = 0.6027, *p* = 0.4451; C22:6 n-3, DHA – HFD effect: F(1,30) = 1.644, *p* = 0.2096, NAC effect: F(1,30) = 26.94, *p* < 0.0001, interaction: F(1,30) = 46.60, *p* < 0.0001; vs. Control group, Table 5). In addition, compared to the HFD group, receiving an HFD with the addition of NAC to rats reduced C16:1, PoA (HFD + NAC: -38.7%, *p* < 0.05, Table 5) and also simultaneous augmented C18:3 n-3, ALA content (HFD + NAC: +34.8%, *p* < 0.05, Table 5) in FFA pool.

**Table 6:** N-acetylcysteine (NAC) influence on the fatty acids composition in phospholipid (PL) fraction in the liver tissue of rats subjected to a standard diet (Control) or a high-fat diet (HFD). The values obtained in gas-liquid chromatography (GLC) method are expressed in nanomoles per gram of tissue and presented as mean ± standard deviation (SD); *n* = 6 rats per group. Statistical analysis was performed using two-way ANOVA, *t*-test or Mann-Whitney U test. A difference was considered statistically significant at *p* < 0.05 and marked as ^*^ - for the comparisons between HFD, NAC and HFD + NAC groups versus Control group and as ^#^ - for the comparison between HFD + NAC group versus HFD group.

		**Control**	**HFD**	**NAC**	**HFD + NAC**
SFA	C14:0, MA	208.51 ± 23.61	228.77 ± 49.53 ^*^	165.37 ± 9.14 ^*^	197.13 ± 26.64
	C16:0, PA	20487.43 ± 2031.97	24874.69 ± 2011.84 ^*^	20473.57 ± 2100.06	23262.70 ± 1139.29
	C18:0, SA	23772.08 ± 4532.06	41687.89 ± 3261.26 ^*^	24311.07 ± 2407.91	38352.39 ± 1378.54
	C20:0	81.99 ± 7.44	113.63 ± 32.27 ^*^	54.46 ± 9.67 ^*^	60.02 ± 14.00 ^* #^
	C22:0, BA	111.77 ± 12.15	220.88 ± 16.66 ^*^	100.80 ± 14.36 ^*^	87.40 ± 15.92 ^* #^
	C24:0, LiA	111.36 ± 30.29	150.86 ± 11.78	138.75 ± 5.06	105.07 ± 18.11 ^* #^
MUFA	C16:1, PoA	620.54 ± 87.77	442.98 ± 49.82 ^*^	745.56 ± 168.36	326.56 ± 55.21^* #^
	C18:1, OA	2479.78 ± 383.96	3896.84 ± 377.45 ^*^	2510.53 ± 213.49	3956.39 ± 405.73
	C24:1, NA	267.88 ± 17.35	259.56 ± 35.29 ^*^	194.76 ± 47.37 ^*^	80.92 ± 14.15 ^* #^
PUFA	C18:2 n-6, LA	18044.08 ± 3244.38	15938.73 ± 351.81 ^*^	17540.32 ± 1736.83	16147.81 ± 1653.35
	C18:3 n-3, ALA	145.53 ± 23.18	168.76 ± 47.55	146.61 ± 17.76 ^*^	125.55 ± 24.38 ^* #^
	C20:5 n-3, EPA	167.64 ± 39.18	359.20 ± 89.97 ^*^	185.84 ± 16.94 ^*^	121.32 ± 16.47 ^* #^
	C22:6 n-3, DHA	4835.01 ± 685.13	10040.38 ± 1552.39 ^*^	5172.12 ± 938.30 ^*^	11974.54 ± 1010.62 ^* #^

SFA - saturated fatty acid; MUFA - monounsaturated fatty acid; PUFA - polyunsaturated fatty acid; HFD - high-fat diet; NAC - n-acetylcysteine.

In PL fraction the level of FA from SFA was changed (C14:0, MA – HFD effect: F(1,28) = 6.312, *p* = 0.0180, NAC effect: F(1,28) = 13.05, *p* = 0.0012, interaction: F(1,28) = 0.3090, *p* = 0.5827; C16:0, PA – HFD effect: F(1,27) = 31.50, *p* < 0.0001, NAC effect: F(1,27) = 1.617, *p* = 0.2144, interaction: F(1,27) = 1.562, *p* = 0.2221; C18:0, SA – HFD effect: F(1,30) = 235.1, *p* < 0.0001, NAC effect: F(1,30) = 1.801, *p* = 0.1897, interaction: F(1,30) = 3.456, *p* = 0.0729; C20:0 – HFD effect: F(1,27) = 10.88, *p* = 0.0027, NAC effect: F(1,27) = 51.78, *p* < 0.0001, interaction: F(1,27) = 5.353, *p* = 0.0285; C22:0, BA – HFD effect: F(1,22) = 74.14, *p* < 0.0001, NAC effect: F(1,22) = 168.9, *p* < 0.0001, interaction: F(1,22) = 121.5, *p* < 0.0001; C24:0, LiA – HFD effect: F(1,23) = 0.1969, *p* = 0.6614, NAC effect: F(1,23) = 1.970, *p* = 0.1738, interaction: F(1,23) = 31.17, *p* < 0.0001; vs. Control group, Table 6). PL’s FA from MUFA and PUFA pools were also changed (C16:1, PoA – HFD effect: F(1,35) = 91.09, *p* < 0.0001, NAC effect: F(1,35) = 0.01890, *p* = 0.8914, interaction: F(1,35) = 14.92, *p* = 0.0005; C18:1, OA – HFD effect: F(1,36) = 177.1, *p* < 0.0001, NAC effect: F(1,36) = 0.1762, *p* = 0.6772, interaction: F(1,36) = 0.01792, *p* = 0.8943; C24:1, NA – HFD effect: F(1,29) = 36.57, *p* < 0.0001, NAC effect: F(1,29) = 155.3, *p* < 0.0001, interaction: F(1,29) = 27.29, *p* < 0.0001; C18:2 n-6, LA– HFD effect: F(1,34) = 7.322, *p* = 0.0106, NAC effect: F(1,34) = 0.05197, *p* = 0.8210, interaction: F(1,34) = 0.3041, *p* = 0.5849; C18:3 n-3, ALA – HFD effect: F(1,27) = 0.01295, *p* = 0.9102, NAC effect: F(1,27) = 4.882, *p* = 0.0358, interaction: F(1,27) = 5.396, *p* = 0.0280; C20:5 n-3, EPA – HFD effect: F(1,33) = 14.85, *p* = 0.0005, NAC effect: F(1,33) = 44.42, *p* < 0.0001, interaction: F(1,33) = 60.36, *p* < 0.0001; C22:6 n-3, DHA – HFD effect: F(1,36) = 339.3, *p* < 0.0001, NAC effect: F(1,36) = 12.14, *p* = 0.0013, interaction: F(1,36) = 6.002, *p* = 0.0193; vs. Control group, Table 6). In the HFD + NAC group we also noted a decline in the PL’s amount of C20:0, C22:0, BA, C24:0, LiA, C16:1, PoA, C24:1, NA, C18:3 n-3, ALA, C20:5 n-3, EPA (HFD + NAC: -47.2%, -60.4%, -30.3%, -26.3%, -68.8%, -25.6%, -66.2%, respectively, *p* < 0.05, Table 6) than in the HFD group. Only PL’s C22:6 n-3, DHA concentration was enhanced in the HFD + NAC group (HFD: +19.3%, vs. HFD group, *p* < 0.05, Table 6).

**Fig. 1:**
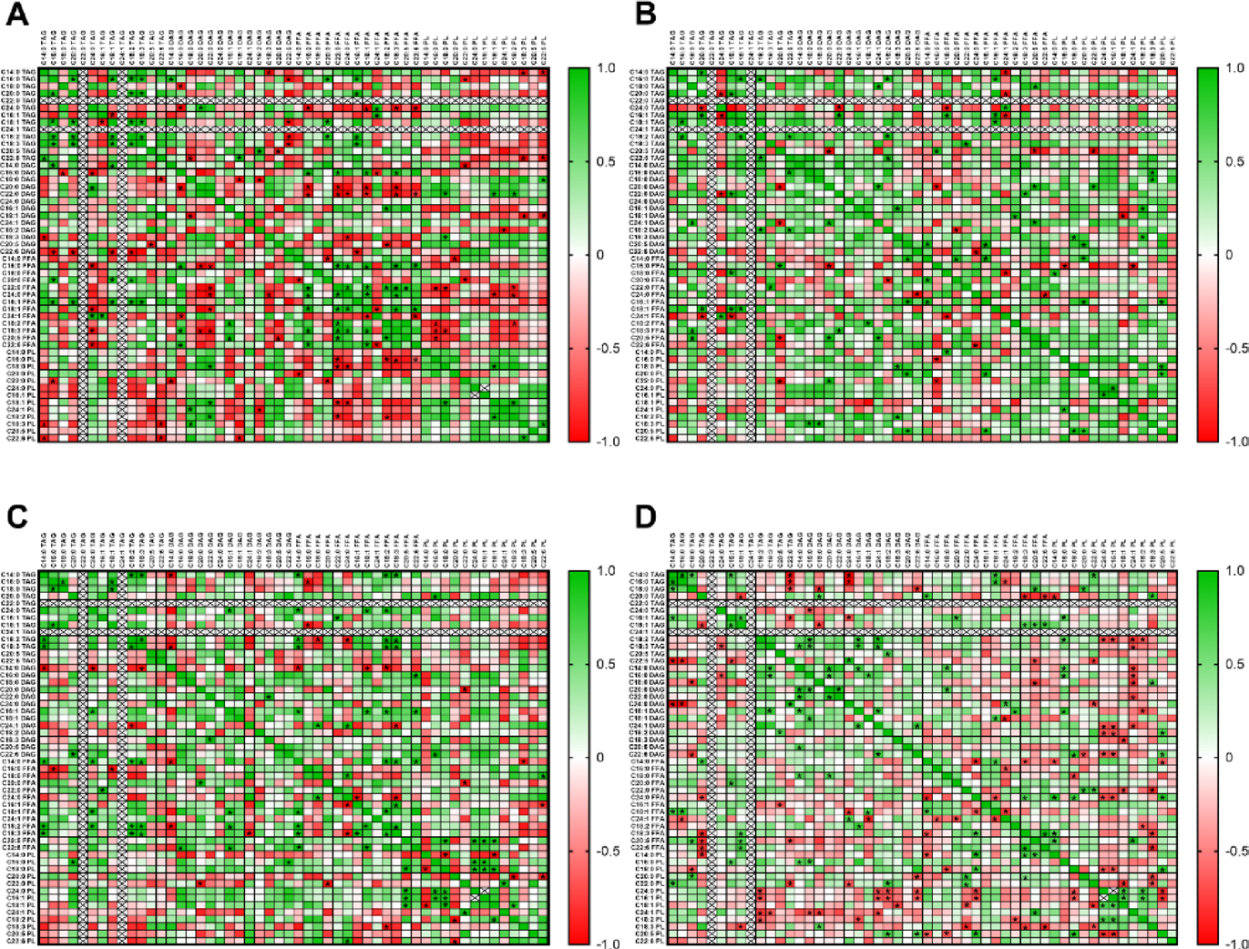
Heat maps of correlation between tested fatty acids composition in triacylglycerol (TAG), diacylglycerol (DAG), free fatty acid (FFA) and phospholipid (PL) fractions in the liver tissue of rats subjected to a standard diet (Control; **A**), a high-fat diet (HFD; **B**), N-acetylcysteine (NAC; **C**) and a high-fat diet with N-acetylcysteine (HFD + NAC; **D**). Statistical analysis was performed using Pearson correlation coefficients and calculated for *n* = 6 rats per group. Positive and negative correlations are expressed as shades of blue and red, respectively. * – statistically significant correlation (*p* < 0.05).

The heat maps (Fig. 1) display the overall distribution of correlation coefficients across the analyzed variables. Most areas exhibit moderate to strong positive correlations, indicated by the dominance of lighter shades. Only a few regions show darker tones, suggesting weaker or negative relationships. The relatively uniform color pattern implies consistent relationships across most variable pairs, without strong outliers or sharply contrasting clusters.

HFD significantly altered individual fatty acids across TAG, DAG, FFA, and PL fractions, including major SFA (C14:0, MA, C16:0, PA, C18:0, SA), MUFA (C16:1, PoA, C18:1, OA), and PUFA (C18:2 n-6, LA, C18:3 n-3, ALA, C20:5 n-3, EPA, C22:6 n-3, DHA). In contrast, NAC effects were variable and fraction-specific, with significant HFD + NAC interactions observed mainly for selected fatty acids such as C20:0, C24:0, LiA, C24:1, NA, EPA, and DHA, indicating selective modulation of lipid remodeling rather than a uniform effect across all parameters.

### Impact of n-acetylcysteine on the hepatic elongation of fatty acid in all examined fractions

**Fig. 2:**
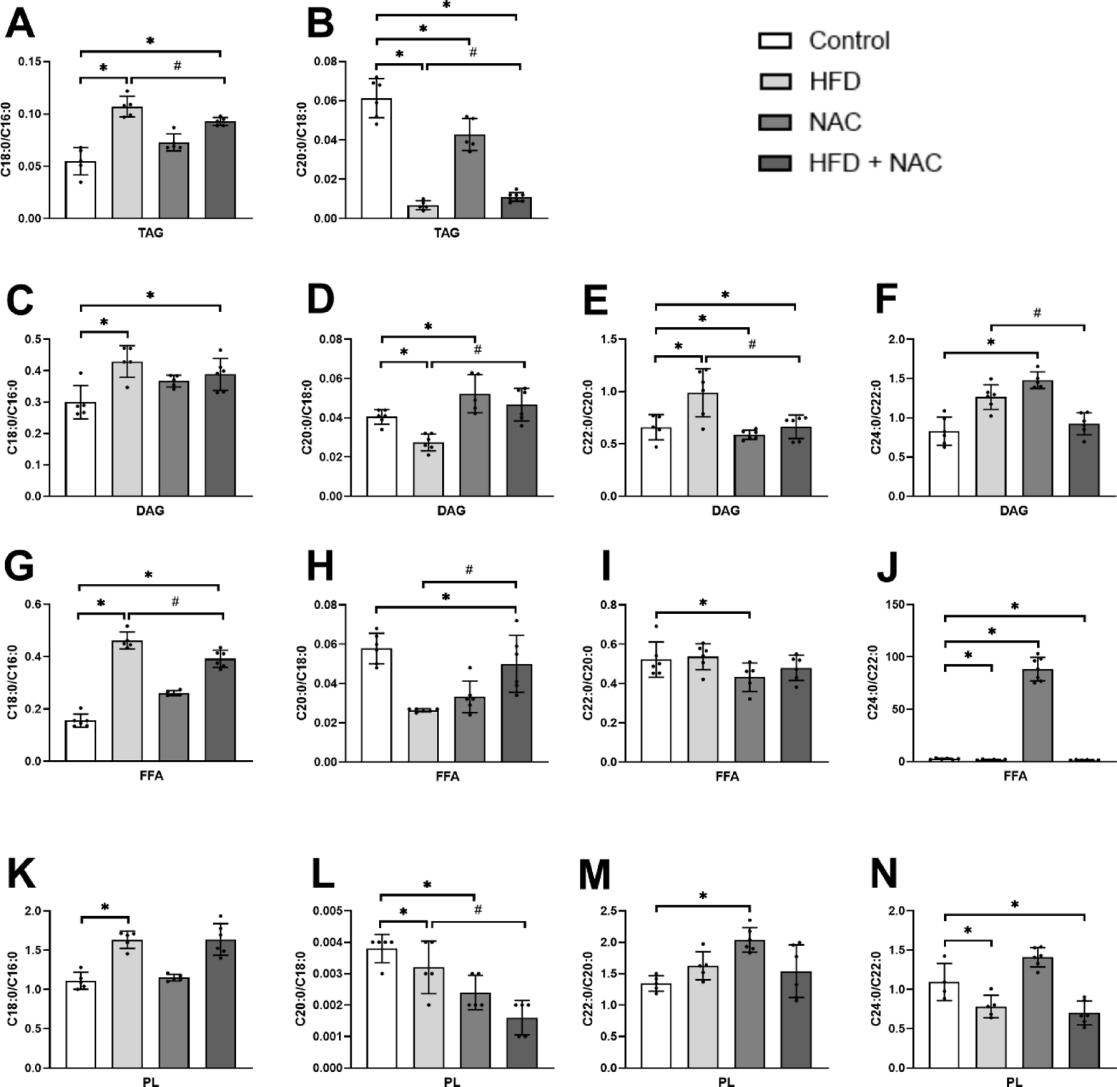
N-acetylcysteine (NAC) influence on the elongation of fatty acid in all examined fractions in the liver tissue of rats subjected to a standard diet (Control) or a high-fat diet (HFD). The values obtained in gas-liquid chromatography (GLC) method are expressed as a ratio data and presented as mean ± standard deviation (SD); *n* = 6 rats per group. Statistical analysis was performed using two-way ANOVA, *t*-test or Mann-Whitney U test. A difference was considered statistically significant at *p* < 0.05 and marked as ^*^ - for the comparisons between HFD, NAC and HFD + NAC groups versus Control group and as ^#^ - for the comparison between HFD + NAC group versus HFD group. TAG – triacylglycerol; DAG – diacylglycerol; FFA – free fatty acid; PL – phospholipid; HFD – high-fat diet; NAC - n-acetylcysteine.HFD significantly affected fatty acid elongation indices (C18:0/C16:0, C20:0/C18:0, C22:0/C20:0, C24:0/C22:0) across TAG, DAG, FFA, and PL fractions. NAC showed selective, fraction-dependent effects with several significant interactions, indicating modulation of elongation pathways in a context-specific manner.

In TAG fraction the ratio of FA elongation were changed under experimental conditions (C18:0/C16:0 – HFD effect: F(1,14) = 57.83, *p* < 0.0001, NAC effect: F(1,14) = 0.1602, *p* = 0.6950, interaction: F(1,14) = 11.57, *p* = 0.0043; C20:0/C18:0 – HFD effect: F(1,17) = 232.6, *p* < 0.0001, NAC effect: F(1,17) = 5.207, *p* = 0.0356, interaction: F(1,17) = 14.40, *p* = 0.0014; vs. Control group, Fig. 2A and B). Similarly, elongation of FA in DAG was also altered (C18:0/C16:0 – HFD effect: F(1,21) = 17.56, *p* = 0.0004, NAC effect: F(1,21) = 0.4172, *p* = 0.5253, interaction: F(1,21) = 10.21, *p* = 0.0044; C20:0/C18:0 – HFD effect: F(1,17) = 9.571, *p* = 0.0066, NAC effect: F(1,17) = 22.21, *p* = 0.0002, interaction: F(1,17) = 1.148, *p* = 0.2990; C22:0/C20:0 – HFD effect: F(1,19) = 8.918, *p* = 0.0076, NAC effect: F(1,19) = 10.42, *p* = 0.0044, interaction: F(1,19) = 4.616, *p* = 0.0448; C24:0/C22:0 – HFD effect: F(1,13) = 0.5119, *p* = 0.4870, NAC effect: F(1,13) = 3.917, *p* = 0.0694, interaction: F(1,13) = 32.57, *p* < 0.0001; vs. Control group, Fig. 2C, D, E and F). The ratio of FA elongation was different also in FFA fraction (C18:0/C16:0 – HFD effect: F(1,15) = 235.6, *p* < 0.0001, NAC effect: F(1,15) = 1.330, *p* = 0.2668, interaction: F(1,15) = 35.43, *p* < 0.0001; C20:0/C18:0 – HFD effect: F(1,17) = 3.208, *p* = 0.0911, NAC effect: F(1,17) = 0.1225, *p* = 0.7307, interaction: F(1,17) = 24.88, *p* = 0.0001; C22:0/C20:0 – HFD effect: F(1,25) = 1.041, *p* = 0.3174, NAC effect: F(1,25) = 5.277, *p* = 0.0303, interaction: F(1,25) = 0.2972, *p* = 0.5905; C24:0/C22:0 – HFD effect: F(1,14) = 190.2, *p* < 0.0001, NAC effect: F(1,14) = 181.4, *p* < 0.0001, interaction: F(1,14) = 183.9, *p* < 0.0001; vs. Control group, Fig. 2G, H, I and J). Lastly, PL fraction, we also observed changes in FA elongation (C18:0/C16:0 – HFD effect: F(1,21) = 90.27, *p* < 0.0001, NAC effect: F(1,21) = 0.3442, *p* = 0.5637, interaction: F(1,21) = 0.02104, *p* = 0.8860; C20:0/C18:0 – HFD effect: F(1,16) = 4.762, *p* = 0.0444, NAC effect: F(1,16) = 23.05, *p* = 0.0002, interaction: F(1,16) = 0.1905, *p* = 0.6684; C22:0/C20:0 – HFD effect: F(1,16) = 0.7283, *p* = 0.4060, NAC effect: F(1,16) = 5.089, *p* = 0.0384, interaction: F(1,16) = 8.917, *p* = 0.0087; C24:0/C22:0 – HFD effect: F(1,16) = 41.28, *p* < 0.0001, NAC effect: F(1,16) = 2.248, *p* = 0.1533, interaction: F(1,16) = 6.153, *p* = 0.0246; vs. Control group, Fig. 2K, L, M and N).

Hepatic C18:0/C16:0 ratio in TAG and FFA was reduced in rats subjected to an HFD and NAC (HFD + NAC: -13.0%, -14.9%, respectively, *p* < 0.05, Fig. 2A and G) compared to the HFD-fed rats. NAC treatment of HFD-fed rats also caused an enlargement in C20:0/C18:0 ratio in TAG, DAG and FFA (HFD + NAC: +64.7%, + 66.1%, + 80.5%, respectively, vs. HFD group, *p* < 0.05, Fig. 2B, D and H). Moreover, the administration of NAC led to a reduction in C20:0/C18:0 ratio in PL (HFD + NAC: -45.9%, *p* < 0.05, Fig. 2L) in relation to the HFD group. High-fat feeding with NAC reduced C22:0/C20:0 ratio and C24:0/C22:0 ratio in DAG (HFD + NAC: -35.9% and − 25.6%, respectively, vs. HFD group, *p* < 0.05, Fig. 2E and F).

### Impact of n-acetylcysteine on the hepatic desaturation of fatty acid in all examined fractions

**Fig. 3:**
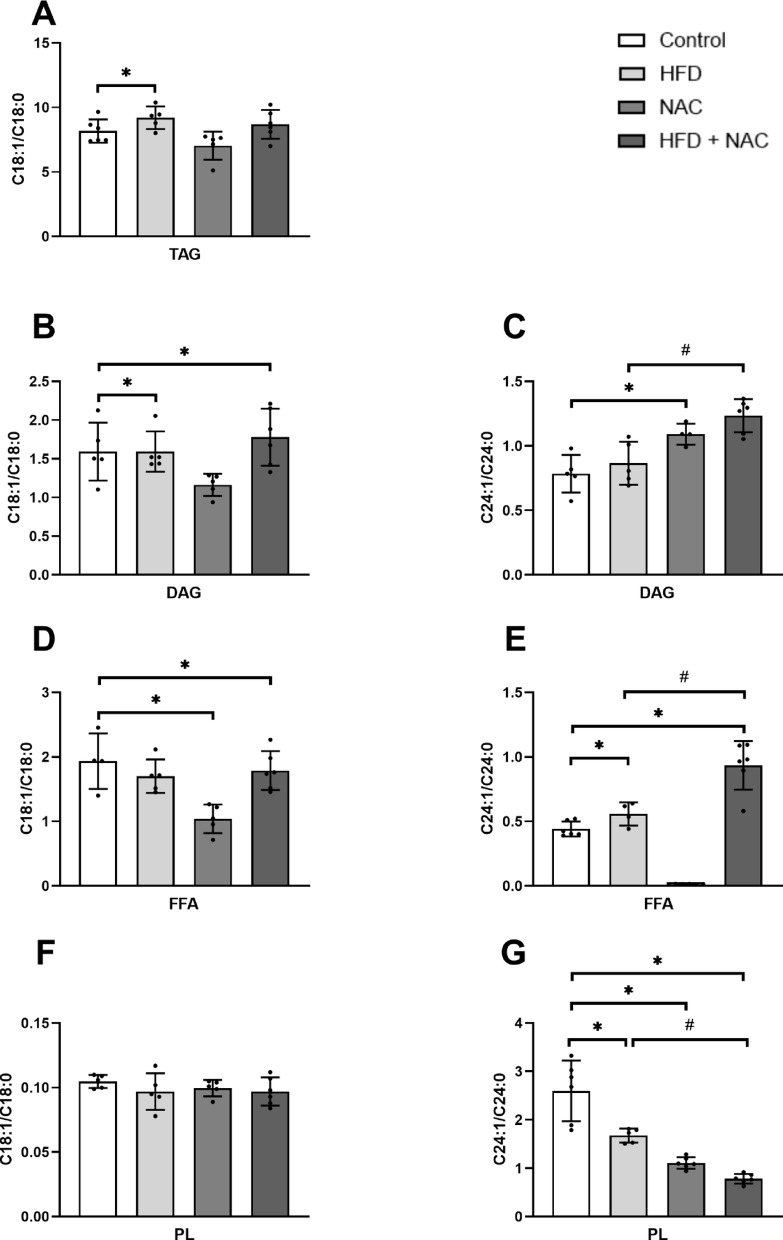
N-acetylcysteine (NAC) influence on the desaturation of fatty acid in all examined fractions in the liver tissue of rats subjected to a standard diet (Control) or a high-fat diet (HFD). The values obtained in gas-liquid chromatography (GLC) method are expressed as a ratio data and presented as mean ± standard deviation (SD); *n*=6 rats per group. Statistical analysis was performed using two-way ANOVA, *t*-test or Mann-Whitney U test. A difference was considered statistically significant at *p* < 0.05 and marked as ^*^ - for the comparisons between HFD, NAC and HFD+NAC groups versus Control group and as ^#^ - for the comparison between HFD+NAC group versus HFD group. TAG – triacylglycerol; DAG – diacylglycerol; FFA – free fatty acid; PL – phospholipid; HFD – high-fat diet; NAC - n-acetylcysteine.

In TAG fraction the ratio of FA desaturation were changed under experimental conditions (C18:1/C18:0 – HFD effect: F(1,32) = 18.34, *p* = 0.0002, NAC effect: F(1,32) = 3.950, *p* = 0.0555, interaction: F(1,32) = 1.698, *p* = 0.2019; vs. Control group, Fig. 3A). Desaturation index in DAG was also altered (C18:1/C18:0 – HFD effect: F(1,21) = 6.955, *p* = 0.0154, NAC effect: F(1,21) = 1.034, *p* = 0.3208, interaction: F(1,21) = 6.884, *p* = 0.0159; C24:1/C24:0 – HFD effect: F(1,16) = 0.6644, *p* = 0.4270, NAC effect: F(1,16) = 12.93, *p* = 0.0024, interaction: F(1,16) = 0.03414, *p* = 0.8557; vs. Control group, Fig. 3B and C). The ratio of FA desaturation was different also in FFA fraction (C18:1/C18:0 – HFD effect: F(1,17) = 3.939, *p* = 0.0636, NAC effect: F(1,17) = 9.309, *p* = 0.0072, interaction: F(1,17) = 14.04, *p* = 0.0016; C24:1/C24:0 – HFD effect: F(1,13) = 61.80, *p* < 0.0001, NAC effect: F(1,13) = 0.1858, *p* = 0.6735, interaction: F(1,13) = 36.96, *p* < 0.0001; vs. Control group, Fig. 3D and E). In PL fraction, we also observed changes in FA desaturation index (C18:1/C18:0 – HFD effect: F(1,21) = 1.334, *p* = 0.2610, NAC effect: F(1,21) = 0.2450, *p* = 0.6257, interaction: F(1,21) = 0.6807, *p* = 0.4186; C24:1/C24:0 – HFD effect: F(1,26) = 26.76, *p* < 0.0001, NAC effect: F(1,26) = 97.26, *p* < 0.0001, interaction: F(1,26) = 6.165, *p* = 0.0198; vs. Control group, Fig. 3F and G).

C24:1/C24:0 index in DAG and FFA was increased in the HFD + NAC group (HFD + NAC: +32.0% and + 66.4%, respectively, *p* < 0.05, Fig. 3C and E) in relation to the HFD group. Moreover, in the same experimental group hepatic C24:1/C24:0 desaturation index in PL fraction was reduced (HFD + NAC: -53.2%, *p* < 0.05, Fig. 3G) than in the HFD group. 

HFD significantly altered fatty acid desaturation indices (C18:1/C18:0 and C24:1/C24:0) across TAG, DAG, FFA, and PL fractions. NAC showed fraction-dependent effects with several significant HFD + NAC interactions, particularly in DAG, FFA, and PL pools, indicating selective modulation of desaturation processes rather than a uniform effect.

### Impact of n-acetylcysteine on the hepatic *de novo* lipogenesis in all examined fractions

**Fig. 4:**
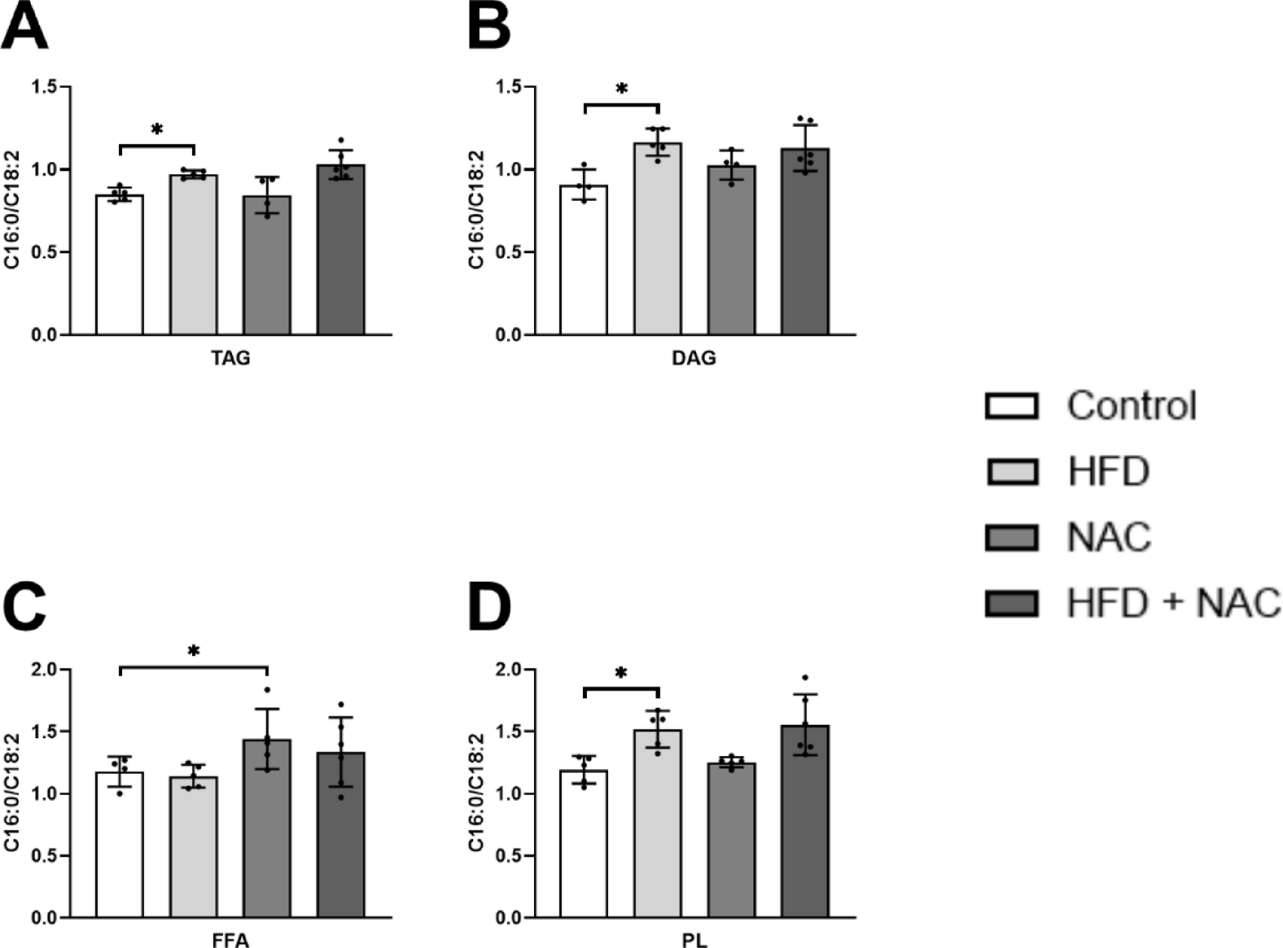
N-acetylcysteine (NAC) influence on the *de novo* lipogenesis all examined fractions in the liver tissue of rats subjected to a standard diet (Control) or a high-fat diet (HFD). The values obtained in gas-liquid chromatography (GLC) method are expressed as a ratio data and presented as mean ± standard deviation (SD); *n* = 6 rats per group. Statistical analysis was performed using two-way ANOVA, *t*-test or Mann-Whitney U test. A difference was considered statistically significant at *p* < 0.05 and marked as ^*^ - for the comparisons between HFD, NAC and HFD + NAC groups versus Control group and as ^#^ - for the comparison between HFD + NAC group versus HFD group. TAG – triacylglycerol; DAG – diacylglycerol; FFA – free fatty acid; PL – phospholipid; HFD – high-fat diet; NAC - n-acetylcysteine.

Hepatic C16:0/C18:2 *de novo* lipogenesis index in all lipid fraction was different after feeding with an HFD and/or NAC (TAG – C16:0/C18:2 – HFD effect: F(1,40) = 54.00, *p* < 0.0001, NAC effect: F(3,40) = 0.5645, *p* = 0.6416, interaction: F(3,40) = 0.7809, *p* = 0.5116; DAG – C16:0/C18:2 – HFD effect: F(1,18) = 16.22, *p* = 0.0008, NAC effect: F(1,18) = 1.632, *p* = 0.2177, interaction: F(1,18) = 1.105, *p* = 0.3071; FFA – C16:0/C18:2 – HFD effect: F(1,19) = 0.07700, *p* = 0.7844, NAC effect: F(1,19) = 6.607, *p* = 0.0187, interaction: F(1,19) = 0.001037, *p* = 0.9747; PL – C16:0/C18:2 – HFD effect: F(1,21) = 20.11, *p* = 0.0002, NAC effect: F(1,21) = 0.3142, *p* = 0.5810, interaction: F(1,21) = 0.1246, *p* = 0.7277; vs. Control group, Fig. 4A, B, C and D).

HFD significantly increased the hepatic C16:0/C18:2 de novo lipogenesis index in TAG, DAG, and PL fractions, while no consistent NAC effect or interaction was observed in these lipid pools. In contrast, FFA showed a significant NAC effect but no HFD effect or interaction, indicating a limited and fraction-specific influence of NAC on this DNL-related index.

### Impact of n-acetylcysteine on the hepatic expression of protein and gene regulated cellular transport of fatty acids in all examined fractions

**Fig. 5:**
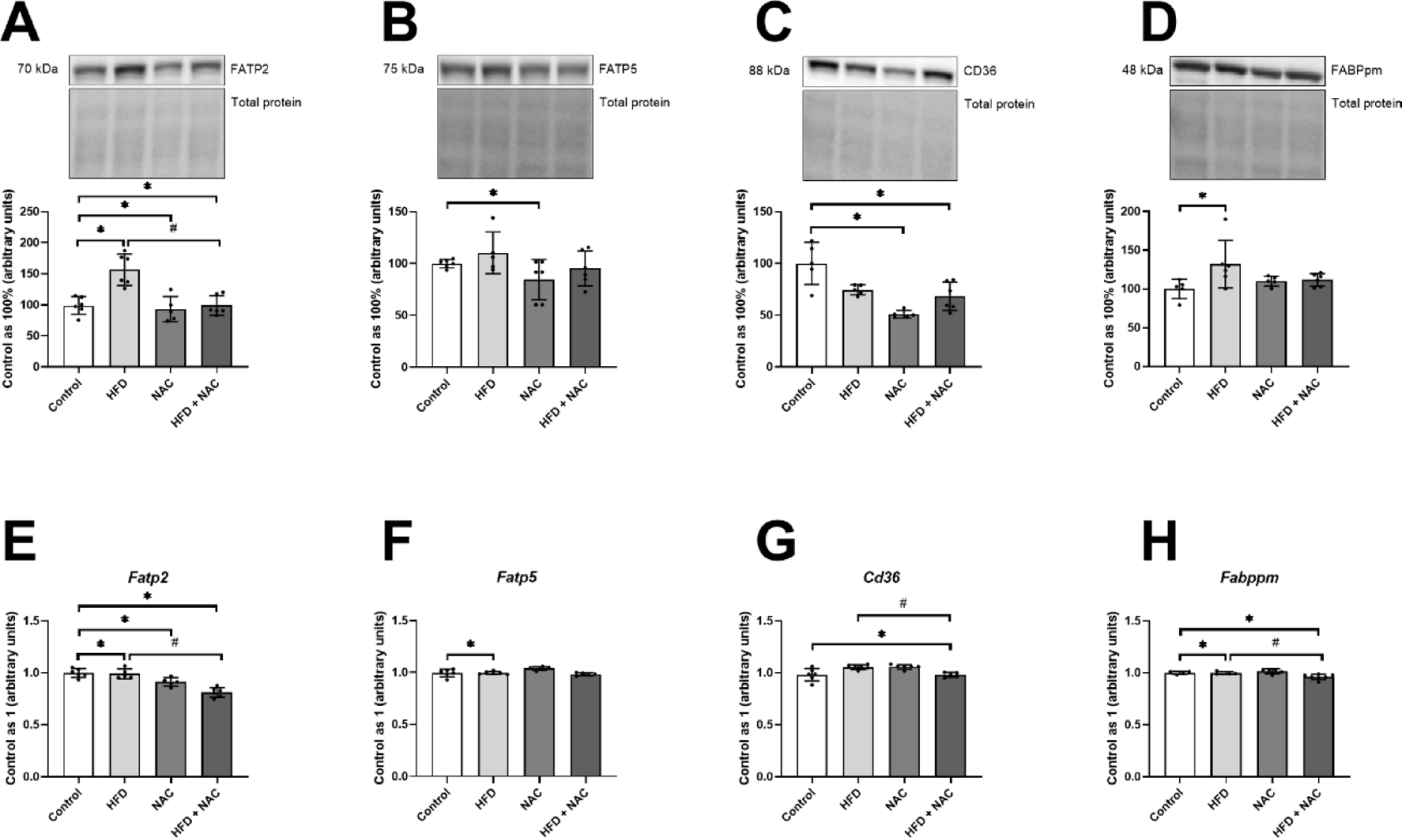
N-acetylcysteine (NAC) influence on the protein (**A**, **B**, **C**, **D**, presented by representative bands as well) and mRNA (**E**, **F**, **G**, **H**) expression of fatty acid transporters in the liver tissue of rats subjected to a standard diet (Control) or a high-fat diet (HFD). The values obtained in Western blot and RT-PCR methods are expressed as fold change in relation to the Control group expressed as 100% or 1, respectively, and presented as mean ± standard deviation (SD); *n* = 6 rats per group. Statistical analysis was performed using two-way ANOVA, *t*-test or Mann-Whitney U test. A difference was considered statistically significant at *p* < 0.05 and marked as ^*^ - for the comparisons between HFD, NAC and HFD + NAC groups versus Control group and as ^#^ - for the comparison between HFD + NAC group versus HFD group. The images of whole gels showing the expression of selected protein and the total protein loading in the liver tissue in the experimental groups are presented in the supplementary data (Figure S1 – S4). FATP2 and FATP5 – fatty acid transport protein 2 and 5; CD36 – fatty acid translocase; FABPpm – membrane-associated fatty acid binding protein; HFD – high-fat diet; NAC – n-acetylcysteine.

Hepatic protein and mRNA expressions of fatty acid transporter 2 and 5 (FATP2 and FATP5) was modified under high-fat feeding and NAC supplementation (FATP2 protein – HFD effect: F(1,19) = 15.51, *p* = 0.0009, NAC effect: F(1,19) = 15.44, *p* = 0.0009, interaction: F(1,19) = 10.26, *p* = 0.0047; *Fatp2* mRNA – HFD effect: F(1,18) = 8.476, *p* = 0.0093, NAC effect: F(1,18) = 48.71, *p* < 0.0001, interaction: F(1,18) = 5.945, *p* = 0.0253; FATP5 protein – HFD effect: F(1,19) = 2.429, *p* = 0.1356, NAC effect: F(1,19) = 5.122, *p* = 0.0355, interaction: F(1,19) = 0.0006255, *p* = 0.9803; *Fatp5* mRNA – HFD effect: F(1,18) = 7.681, *p* = 0.0126, NAC effect: F(1,18) = 2.258, *p* = 0.1503, interaction: F(1,18) = 7.968, *p* = 0.0113; vs. Control group, Fig. 5A, E, B and F). Expression of other transporters like fatty acid translocase (CD36) and membrane-associated fatty acid binding protein (FABPpm) was also altered under treatments (CD36 protein – HFD effect: F(1,17) = 0.5175, *p* = 0.4817, NAC effect: F(1,17) = 24.34, *p* = 0.0001, interaction: F(1,17) = 14.80, *p* = 0.0013; *Cd36* mRNA – HFD effect: F(1,19) = 0.007679, *p* = 0.9311, NAC effect: F(1,19) = 4.392e-005, *p* = 0.9948, interaction: F(1,19) = 23.69, *p* = 0.0001; FABPpm protein – HFD effect: F(1,18) = 4.804, *p* = 0.0418, NAC effect: F(1,18) = 0.4354, *p* = 0.5177, interaction: F(1,18) = 3.914, *p* = 0.0634; *Fabppm* mRNA – HFD effect: F(1,16) = 8.241, *p* = 0.0111, NAC effect: F(1,16) = 1.283, *p* = 0.2741, interaction: F(1,16) = 6.995, *p* = 0.0177; vs. Control group, Fig. 5C, G, D and H).

Hepatic protein and mRNA expression of FATP2 were decreased after NAC supplementation to obese rats (HFD + NAC: -36.7% and − 18.3%, vs. HFD group, *p* < 0.05, Fig. 5A and E). Although mRNA *Cd36* and *Fabppm* were also significantly reduced in the HFD + NAC group (HFD + NAC: -7.3% and − 3.7%, *p* < 0.05, Fig. 5G and H) than in the HFD group.

**Fig. 6:**
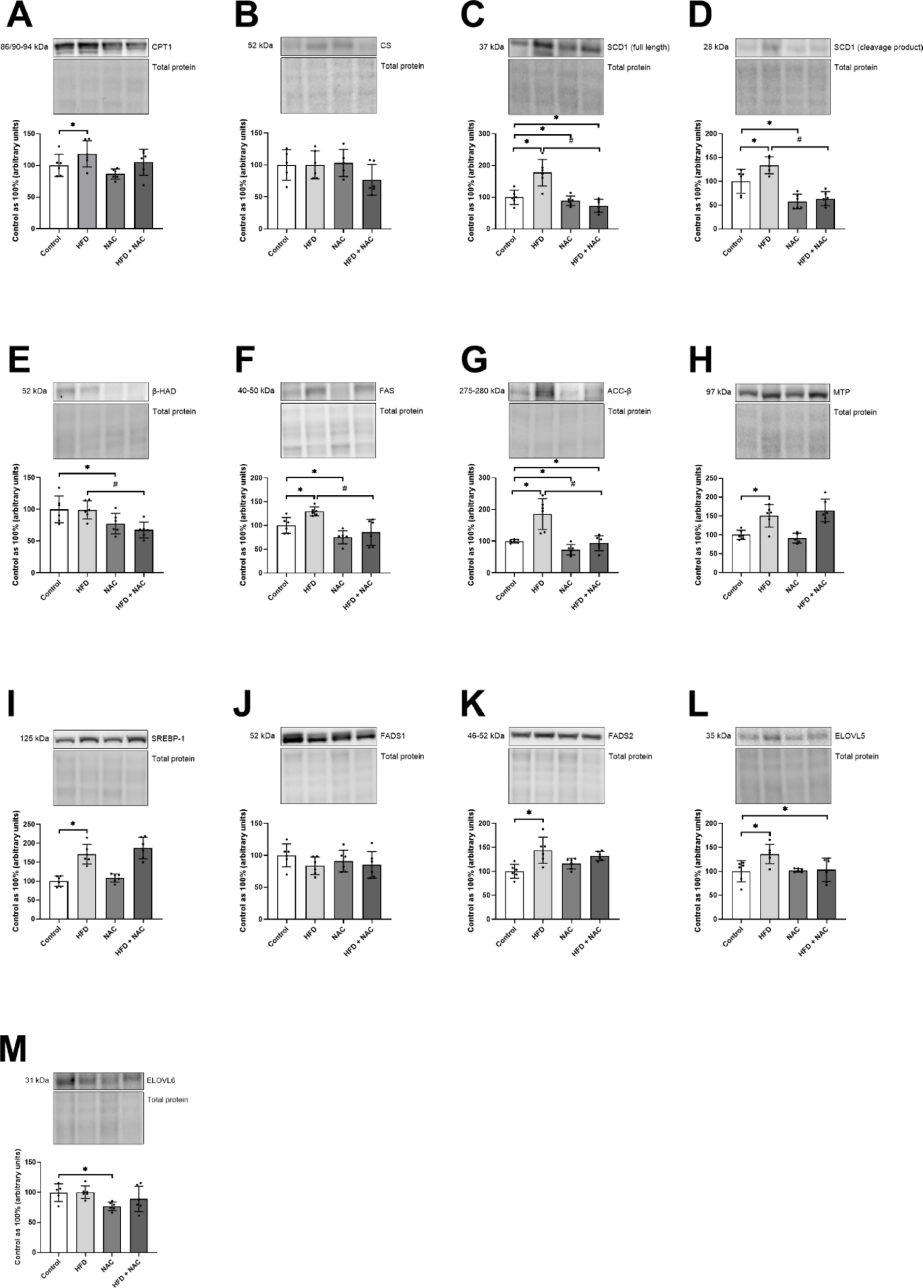
N-acetylcysteine (NAC) influence on the expression of proteins (presented by representative bands as well) regulated lipid metabolism in the liver tissue of rats subjected to a standard diet (Control) or a high-fat diet (HFD). The values obtained in Western blot method are expressed as fold change in relation to the Control group expressed as 100% and presented as mean ± standard deviation (SD); *n* = 6 rats per group. Statistical analysis was performed using two-way ANOVA, *t*-test or Mann-Whitney U test. A difference was considered statistically significant at *p* < 0.05 and marked as ^*^ - for the comparisons between HFD, NAC and HFD + NAC groups versus Control group and as ^#^ - for the comparison between HFD + NAC group versus HFD group. The images of whole gels showing the expression of selected protein and the total protein loading in the liver tissue in the experimental groups are presented in the supplementary data (Figure S5 – S16). CPT1 – mitochondrial carnitine palmitoyltransferase 1; SCD1 – stearoyl-CoA desaturase 1; MTP – microsomal triglyceride transfer protein; CS – citrate synthase; β-HAD – hydroxyacyl-CoA dehydrogenase trifunctional multienzyme complex subunit beta; FAS – fatty acid synthase; ACC-β – acetyl-CoA carboxylase 2; FADS1 and FADS2 – fatty acid desaturase 1 and 2; SREBP1 – sterol regulatory element-binding transcription factor 1; ELOVL5 and ELOVL6 – elongation of very long-chain fatty acids 5 and 6. HFD – high-fat diet; NAC – n-acetylcysteine.

A high-fat diet alone and/in the combination with NAC caused a changes in the expression of mitochondrial carnitine palmitoyltransferase 1 (CPT1) and citrate synthase (CS) (CPT1 – HFD effect: F(1,20) = 6.708, *p* = 0.0175, NAC effect: F(1,20) = 3.573, *p* = 0.0733, interaction: F(1,20) = 0.001326, *p* = 0.9713; CS – HFD effect: F(1,18) = 1.832, *p* = 0.1926, NAC effect: F(1,18) = 1.075, *p* = 0.3135, interaction: F(1,18) = 1.835, *p* = 0.1923; vs. Control group, Fig. 6A and B). Expression of both full length and cleavage product of stearoyl-CoA desaturase 1 (SCD1) was modified in this experiment (SCD1 (full length) – HFD effect: F(1,20) = 7.878, *p* = 0.0109, NAC effect: F(1,20) = 27.19, *p* < 0.0001, interaction: F(1,20) = 17.86, *p* = 0.0004; SCD1 (cleavage product) – HFD effect: F(1,19) = 6.299, *p* = 0.0213, NAC effect: F(1,19) = 51.43, *p* < 0.0001, interaction: F(1,19) = 3.128, *p* = 0.0930; vs. Control group, Fig. 6C and D). Additionally, expression of hydroxyacyl-CoA dehydrogenase trifunctional multienzyme complex subunit beta (β-HAD), fatty acid synthase (FAS), acetyl-CoA carboxylase 2 (ACC-β), microsomal triglyceride transfer protein (MTP) and sterol regulatory element-binding transcription factor 1 (SREBP1) were altered upon experimental treatments with an HFD and/with/or NAC (β-HAD – HFD effect: F(1,20) = 0.6815, *p* = 0.4188, NAC effect: F(1,20) = 16.64, *p* = 0.0006, interaction: F(1,20) = 0.4749, *p* = 0.4987; FAS – HFD effect: F(1,20) = 7.393, *p* = 0.0132, NAC effect: F(1,20) = 22.00, *p* = 0.0001, interaction: F(1,20) = 1.687, *p* = 0.2088; ACC-β – HFD effect: F(1,20) = 21.16, *p* = 0.0002, NAC effect: F(1,20) = 26.55, *p* < 0.0001, interaction: F(1,20) = 7.788, *p* = 0.0113; MTP – HFD effect: F(1,19) = 40.56, *p* < 0.0001, NAC effect: F(1,19) = 0.08686, *p* = 0.7714, interaction: F(1,19) = 1.416, *p* = 0.2487; SREBP1 – HFD effect: F(1,20) = 76.36, *p* < 0.0001, NAC effect: F(1,20) = 2.083, *p* = 0.1644, interaction: F(1,20) = 0.2243, *p* = 0.6409; vs. Control group, Fig. 6E, F, G, H and I). Moreover, fatty acid desaturase 1 and 2 (FADS1 and FADS2) expression were different in all groups (FADS1 – HFD effect: F(1,20) = 2.394, *p* = 0.1375, NAC effect: F(1,20) = 0.2868, *p* = 0.5982, interaction: F(1,20) = 0.5268, *p* = 0.4764; FADS2 – HFD effect: F(1,20) = 18.58, *p* = 0.0003, NAC effect: F(1,20) = 0.07664, *p* = 0.7847, interaction: F(1,20) = 3.980, *p* = 0.0598; vs. Control group, Fig. 6J and K). Elongation of very long-chain fatty acids 5 and 6 (ELOVL5 and ELOVL6) expression were changed in all experimental groups (ELOVL5 – HFD effect: F(1,20) = 5.518, *p* = 0.0292, NAC effect: F(1,20) = 3.818, *p* = 0.0648, interaction: F(1,20) = 4.905, *p* = 0.0385; ELOVL6 – HFD effect: F(1,19) = 1.206, *p* = 0.2859, NAC effect: F(1,19) = 8.010, *p* = 0.0107, interaction: F(1,19) = 0.9178, *p* = 0.3501; vs. Control group, Fig. 6L and M).

Immunoblotting revealed a reduction in full length and cleavage product of SCD1 expression under an HFD with NAC conditions (HFD + NAC: -57.6% and − 52.9%, vs. HFD group, *p* < 0.05, Fig. 6C and D). A decrease in the expression of β-HAD, FAS and ACC-β in rats from the HFD + NAC group was also observed (HFD + NAC: -32.0%, -34.0% and − 49.5%, *p* < 0.05, Fig. 6E, F and G) than in the HFD group.

HFD significantly altered the expression of key genes and proteins involved in fatty acid uptake, synthesis, oxidation, elongation, and desaturation, including CPT1, SCD1, FAS, ACC-β, SREBP1, FADS2, ELOVL5, ELOVL6, and MTP. NAC showed selective and pathway-specific effects, with significant modulation of SCD1, ACC-β, β-HAD, FAS, and ELOVL6, along with several significant interactions, indicating that its influence on hepatic lipid metabolism is not uniform but depends on the specific metabolic pathway and context.

## Discussion

The liver is the key organ involved in lipid metabolism. Systemic disturbances noticed in MASLD are especially characterized by overall triacylglycerol lipid accumulation, which was confirmed in our previous study^17^. It is known that during hepatosteatosis also the composition of lipid fractions is also subject to change. The present data are based on the same experimental model described in our previous publications^14,15,17,19^, in which a single comprehensive experiment was conducted. Liver tissue obtained from this experiment was used for different types of biochemical and molecular analyses. The herein study complements those earlier reports by describing the hepatic fatty acid composition in each lipid pool and, most importantly influence of one of the most used antioxidants, NAC, in the lipid overload conditions. This study investigates how N-acetylcysteine (NAC), an antioxidant, influences lipid metabolism in the liver under conditions of fat overload.

In MASLD, excessive fatty acid uptake and altered lipid composition contribute to steatosis, with key transporters (FATP2, CD36, FABPpm) playing central roles. NAC supplementation reduced the expression of these transporters, suggesting a positive effect against lipid accumulation. It also modulated fatty acid composition by promoting the conversion of saturated fatty acids (C16:0, PA, C18:0, SA) to unsaturated forms via desaturation and elongation processes, potentially lowering inflammation and progression to steatohepatitis. Generally, hepatic lipid metabolism is regulated by the uptake of fatty acids and also their intracellular fate, through the β-oxidation, esterification and export in the form of very low-density lipoproteins (VLDL). Under high dietary intake, an excess of fatty acids is absorbed and driven to hepatocytes due to the presence of specific transporters like fatty acid transport proteins (FATP) and plasma membrane fatty acid-binding protein (FABPpm) being expressed in the liver. When the capacity for β-oxidation and lipid export is exceeded, lipids accumulate within hepatocytes in the form of triglycerides, leading to steatosis. Under high-fat feeding, lipid overload promotes lipotoxicity through the accumulation of harmful lipid intermediates, such as ceramides and diacylglycerols, which impair insulin signaling and exacerbate metabolic dysfunction. This is accompanied by increased oxidative stress and activation of inflammatory pathways, further driving liver injury and MASLD progression^20^. High-fat diet not only cause fatty liver and disturbances in adipose tissue but also lead to systemic metabolic issues that affect various organs, including the brain^21^. NAC may exert additional positive effects by improving redox balance, enhancing antioxidant defenses, and supporting mitochondrial function, thereby mitigating the detrimental effects of lipid excess in the liver. Clinical study showed that CD36 was upregulated in patients with NAFL/NASH in relation to healthy volunteers, which suggests a possible role in the excessive lipid accumulation^22^. These observations are consistent with the present results, which show increasing mRNA expression of *Cd36* after high-fat feeding. Data suggest that hepatic overabsorption of FA could play a role in the initiation and development of simple steatosis^23^. In the herein study, an enhanced hepatic expression of FATP2 (protein) was observed. Prominently, the supplementation of NAC significantly reduced the expression of mentioned transporters, FATP2 (mRNA and protein), *Cd36* (mRNA), and *Fabppm* (mRNA) as well, suggesting a positive antioxidant effect on high availability of dietary fats at an early stage of their transport to the liver. Mice subjected to a Western-type diet and knocked down CD36 were protected from hepatic steatosis and accompanying insulin resistance, which suggests that increased FA uptake constitutes an important point for TAG overload and fatty liver occurrence^23,24^. Falcon et al. describe that liver-specific FATP2 knockdown is able to reduce hepatosteatosis during high-fat feeding by lowering LCFA β-oxidation^25^. Moreover, hepatic CD36 disruption improves steatosis by limiting FA intake and lowering its accumulation inside the hepatocytes^26^. *Cd36* mRNA expression was slightly increased, and the corresponding protein level assessed by Western blot was slightly decreased, but did not reach statistical significance. This indicates that the transcriptional upregulation of *Cd36* was not translated into a measurable increase in protein expression, which represents the functional substance. Importantly, after NAC administration, CD36 protein levels showed a decreasing tendency, suggesting that NAC may attenuate CD36 protein expression despite elevated transcript levels. This discrepancy highlights the importance of evaluating both transcriptional and post-transcriptional regulation when interpreting changes in transporter expression. Although the global FABPpm knockout model is not suitable for the liver tissue (non-specific to liver) to examine the protective effect of some agents, Mukai et al. revealed increased hepatic VLDL secretion in FABPpm-deficient mice^27^. We suspect that a pharmacological agent like NAC, which attenuates the expression of all FA transporters, i.e., FATP2 and CD36, FABPpm, can limit hepatocytes from lipid overload and protect from steatosis development.

Lipids accumulation in the liver is caused by a diet rich in fat, enhanced lipolysis by lipid droplets due to insulin resistance in adipose tissue, elevated production of lipid (de novo lipogenesis) and then stored within lipid droplets as well as changes in the secretion of VLDL. An index of *de novo* lipogenesis (DNL ratio), calculated from fatty acid composition, was used as an indirect marker of hepatic lipogenic activity. Our results revealed the enhanced DNL ratio in TAG, DAG and PL fraction in rats fed with an HFD. This process contributes to excessive hepatic accumulation of fatty acids and triglycerides, thereby promoting steatosis development. Persistent activation of *de novo* lipogenesis, together with impaired fatty acid oxidation, exacerbates lipid overload in the liver and promotes metabolic inflammation^28^. The lack of effect of NAC on the DNL ratio, which was observed in the herein study, suggests that its actions are likely independent of direct modulation of lipogenic pathways. Instead, NAC may exert its beneficial effects primarily through the attenuation of oxidative stress and improvement of FA oxidation.

The cellular FA diffusion determines the subsequent β-oxidation in the mitochondria or TAG formation being stored in lipid droplets by esterification pathways. Under the oxidation process, FA can be used to create the fatty acid molecules with divergent chain lengths, numbers of double bonds, and also desaturation degrees^6^. Availability data clearly describe that in the NAFLD condition, FA are prone to oxidize into saturated fatty acid (SFA) pools. Our results revealed increased SFA content in TAG and PL fractions in rats with steatosis (confirmed by histological analysis in our previous publications^17,19^, which clearly suggests the role of saturated fatty acids in the lipid deposition. Desaturation and elongation processes are essential in fatty acid metabolism that influence the production of long-chain and PUFA, which are crucial for cell membrane structure and lipid signaling. These reactions are primarily facilitated by enzymes such as SCD1 and the desaturases Δ5- and Δ6-desaturase (FADS1 and FADS2), as well as the elongation of very long-chain fatty acids (ELOVL) enzymes. These processes mainly occur in the endoplasmic reticulum of metabolically active tissues, particularly the liver, with additional involvement from adipose tissue and the brain^29,30^. The functioning of these pathways is influenced by the types of dietary fats, insulin signaling, and levels of inflammation. SFA and MUFA can reduce desaturase activity, while nutritional and hormonal signals can increase enzyme expression and the rate of these pathways. Conversely, PUFA may inhibit the earlier steps of desaturation and elongation, thus affecting lipid balance^31^. Disruption of these processes has been linked to metabolic disorders, such as hepatic steatosis, where changes in enzyme activity lead to abnormal lipid buildup and alterations in membrane structure. The main FA from all SFA groups, palmitic acid (C16:0, PA) and stearic acid (C18:0, SA), can be converted in the next step to MUFA by SCD1^6^. In the present data, we noticed higher expression of SCD1 in the HFD group, which resulted in higher MUFA concentration in TAG. Our study also revealed significant changes in the desaturation index of FA in selected lipid fractions after NAC administration to HFD-fed rats. It was seen in the increase of the C24:1/C24:0 ratio, especially in DAG and FFA fractions. Mentioned changes are an indicator of the process of double bond introduction into the FA structure. Specifically, liver tissue is primarily involved in the conversion of SFA to MUFA and PUFA, impacting lipid composition and cellular processes. Desaturases enzymes modify FA for their incorporation into glycerolipids and are then secreted as lipoprotein, which reveals hepatic positive effect in response to lipid overload^32^. So, we suspect that NAC, by increasing C24:1/C24:0 desaturation index, may protect the liver by converting SFA to UFA, which is finally secreted as lipoprotein, especially VLDL.

Among the SFA pool, we also observed a higher content of C16:0, PA and C18:0, SA, especially in the TAG fraction, promoting hepatic lipid accumulation, steatosis development, and its progression to steatohepatitis. Kessler et al. showed that enhanced C16:0, PA and C18:0, SA levels contribute to the inflammation occurrence in early hepatocarcinogenesis^33^. It is consistent with the augmentation of C18:0/C16:0 elongation index in our study, which was enhanced in obese rats subjected to an HFD (body mass previously published^15^. In a work by Kessler et al. enhanced C18:0/C16:0 ratio was noticed in a murine model of NASH^33^. Data conducted by Tang et al. showed that different concentrations (200–600 mg/kg) of dietary GSH reduced hepatic TAG content, limiting HFD-induced steatosis^34^. Decreased elongation of C18:0, SA to C20:0 is related to higher synthesis of bioactive lipids like C20:5 n-3, EPA and C22:6 n-3, DHA, which have anti-inflammatory and pro-resolving properties^35^. In particular, C20:5 n-3, EPA and C22:6 n-3, DHA, concentration was enhanced in the TAG fraction, the main lipid pool deposited in hepatocytes under steatosis occurrence. Moreover, in the herein study, NAC supplementation to rats fed with an HFD also induced an increase in the elongation ratio of C20:0/C18:0 in almost all lipid fractions, except PL, and a decrease in C22:0/C20:0 and C24:0/C22:0 ratios in DAG. These observations suggest that by decreasing the ratio of very-long chain fatty acid (VLCFA), NAC may limit the development of steatosis and disruption in elongation processes, which was confirmed by immunoblotting. It was a non-significant effect of NAC on ELOVL5 and ELOVL6 expression, which cannot be related to the direct target of the PPAR pathway^36^.

The choice of N-acetylcysteine (NAC) as a dietary supplement is justified by its known antioxidant properties and protective potential. The focus on fatty acid transport and metabolism, rather than just the overall degree of steatosis, distinguishes this work from many studies on NAC. Nevertheless, our study has some limitations. The model is limited to male Wistar rats. The lack of sex analysis and the use of other models (e.g., genetically modified mice) limit the generalizability of the results. However, no tests of liver function (e.g., ALT, AST) were performed that would be clinically relevant.

**Scheme 1:**
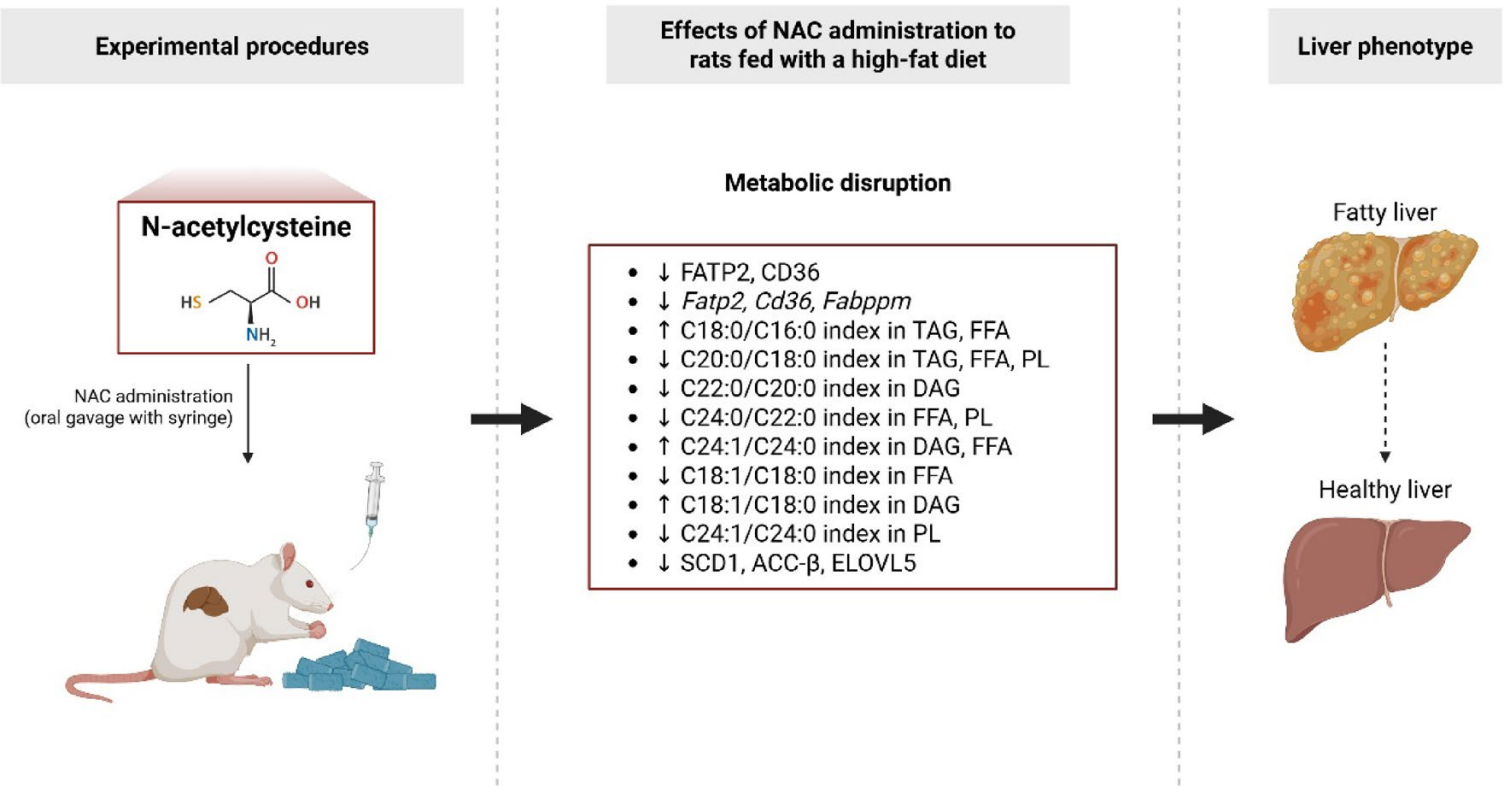
Data summarizes the effects of n-acetylcysteine on the lipid metabolism in rats with steatosis induced by a high-fat diet. Created with BioRender.com.

## Conclusions

In conclusion, this study presents the positive effect of N-acetylcysteine on the limitation of lipid deposition in rats receiving a high-fat diet. We demonstrated that NAC decreased fatty acid transport into hepatocytes, which is the first line of defense against enhanced lipid availability and provides insights into hepatocyte limits the development steatosis. There was a decrease in protein and/or mRNA expression of FATP2, FABPpm, and CD36 in the HFD + NAC group. Supplementation of NAC also significantly reduced elongation of C16:0, PA to C18:0, SA, that process is often upregulated in steatosis. Additionally, an enlarged C20:0/C18:0 elongation ratio with simultaneous enhancement in C20:5 n-3, EPA and C22:6 n-3, DHA levels after treatment with NAC points anti-inflammatory and pro-resolving properties of this target, suggesting its potential role in the limitation the development of simple steatosis changes. NAC may limit MASLD development by modulating hepatic lipid metabolism, including fatty acid transport and remodeling, rather than acting only as an antioxidant. It improves lipid composition by increasing PUFA, potentially reducing lipotoxicity and inflammation. Overall, the study supports the concept that early metabolic interventions can shift hepatic lipid handling toward a less lipotoxic state (Scheme 1). However, translation to humans requires caution, as dose, bioavailability, and long-term effects of NAC in steatosis are not fully established.

## Supplementary Information

Below is the link to the electronic supplementary material.

Supplementary Material 1

Supplementary Material 2

Supplementary Material 3

## Data Availability

All data generated and analyzed during this study are included in this published article and its supplementary information files.

## Declarations

### Methods statement

All methods are reported in accordance with ARRIVE guidelines and carried out in accordance with relevant guidelines and regulations.
