# Supplementary material for: New approaches of N-acetylcysteine on fatty acid transport and metabolism in a rat model of MASLD induced by high-fat diet
Source: Sci Rep. 2026 May 31;16:19570. doi: 10.1038/s41598-026-55583-w (PMC13294378; doi:10.1038/s41598-026-55583-w)
Supplement: Supplementary file 2 — Supplementary Material 2 [file 41598_2026_55583_MOESM2_ESM.pdf]

MEDICAL UNIVERSITY OF BIALYSTOK

Bialystok, November 21st 2025

Ethics Declaration:

All animal experiments were performed in accordance with institutional, national, and international guidelines and regulations for the care and use of laboratory animals. Experimental protocols were reviewed and approved by the Local Ethical Committee for Animal Experiments at the Medical University of Bialystok (Approval No. 21/2017).

KIEROWNIK
Zakładu Fizjologii
prof. dr hab. Adrian Chabowski
