# Supplementary material for: New approaches of N-acetylcysteine on fatty acid transport and metabolism in a rat model of MASLD induced by high-fat diet
Source: Sci Rep. 2026 May 31;16:19570. doi: 10.1038/s41598-026-55583-w (PMC13294378; doi:10.1038/s41598-026-55583-w)

Supporting data – Western blots

Loading order in all proteins: Control, High-FatDiet, N-acetylcysteine, High-FatDiet + N-acetylcysteine.

FATP2

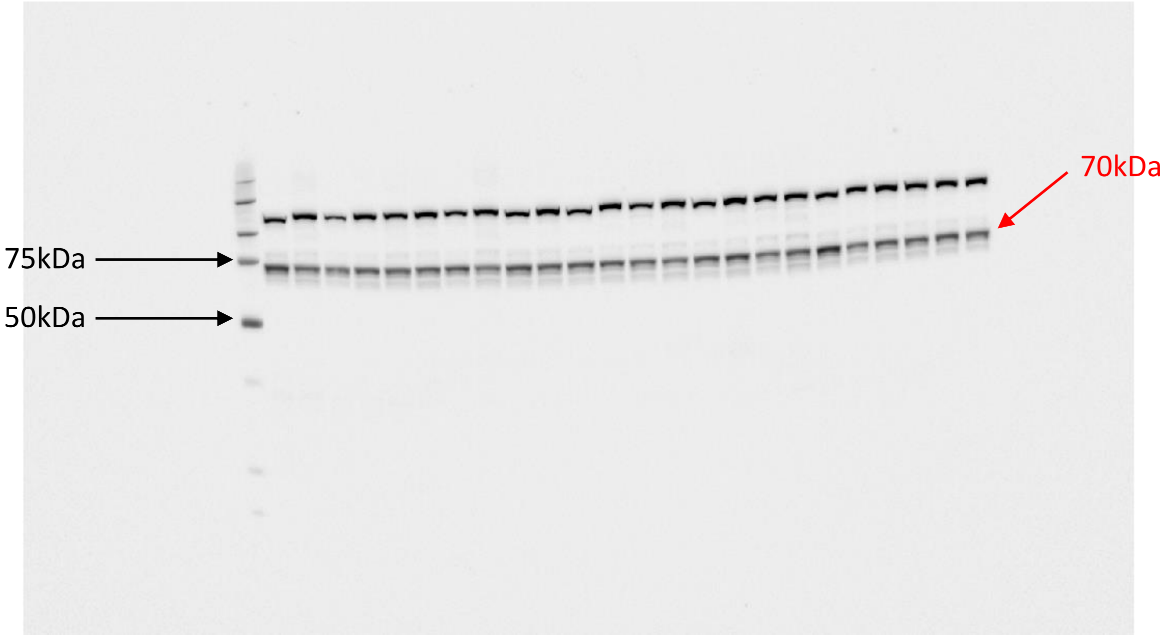

Total protein

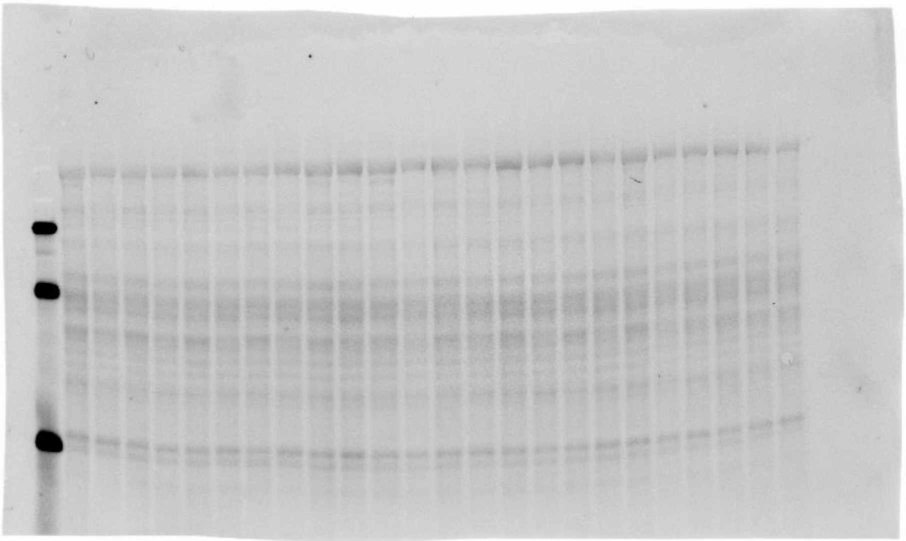

FATP5

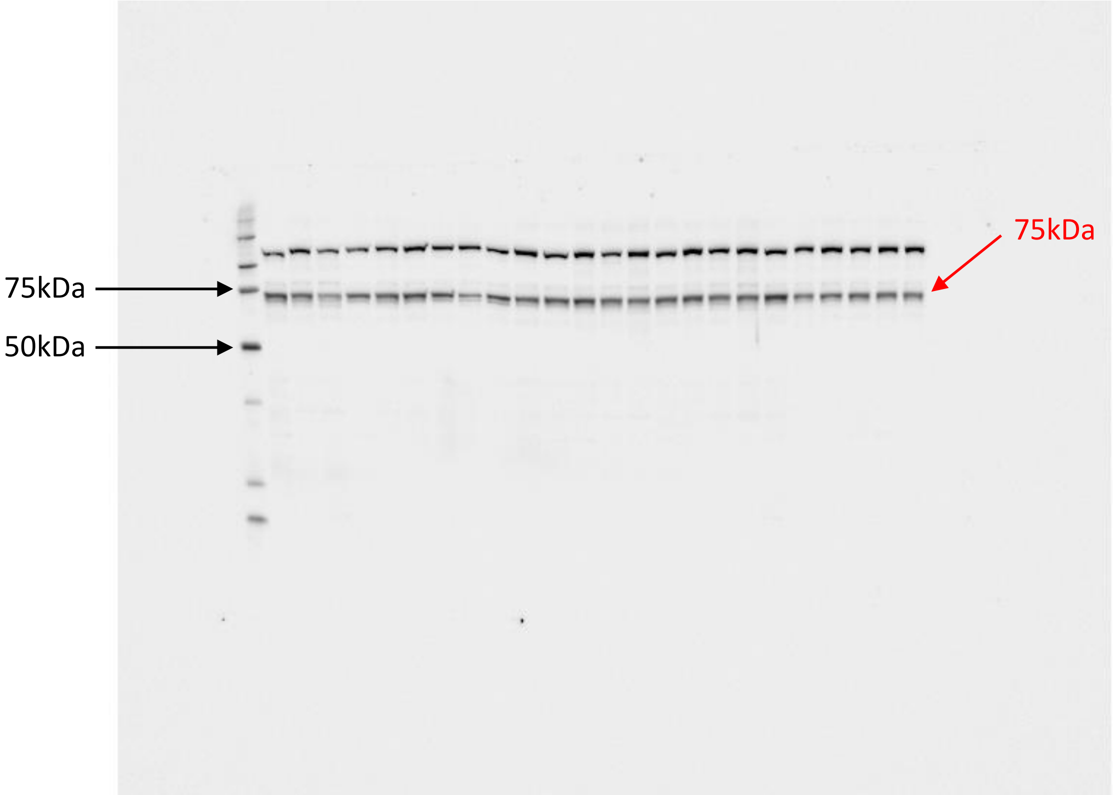

Total protein

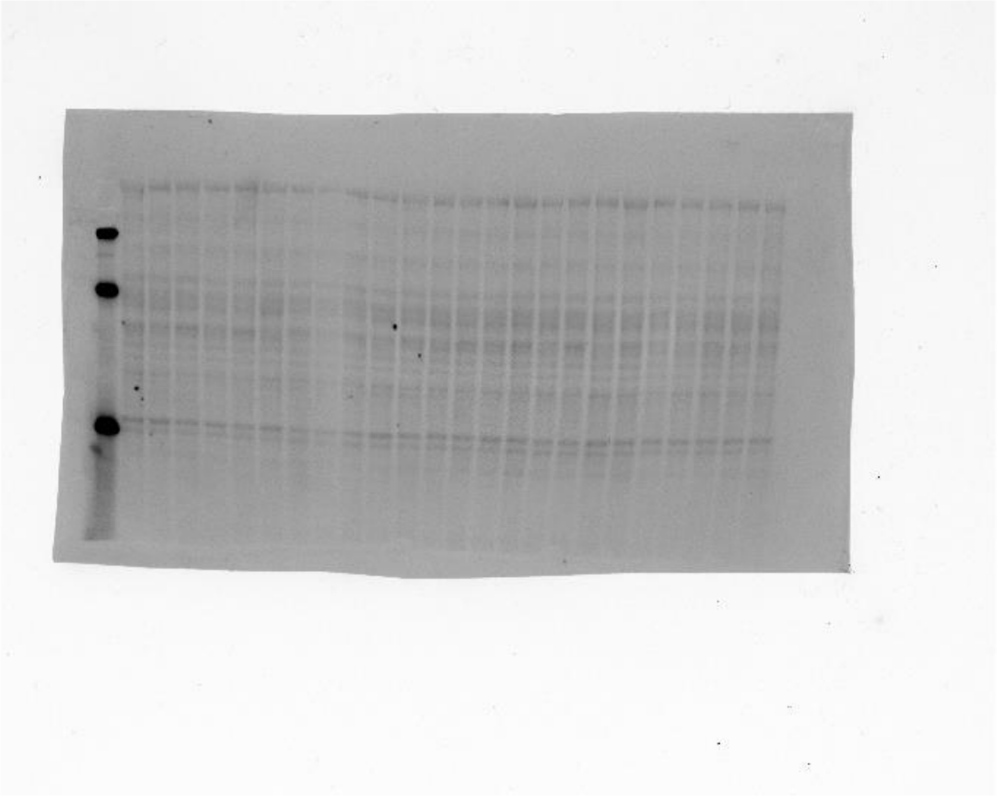

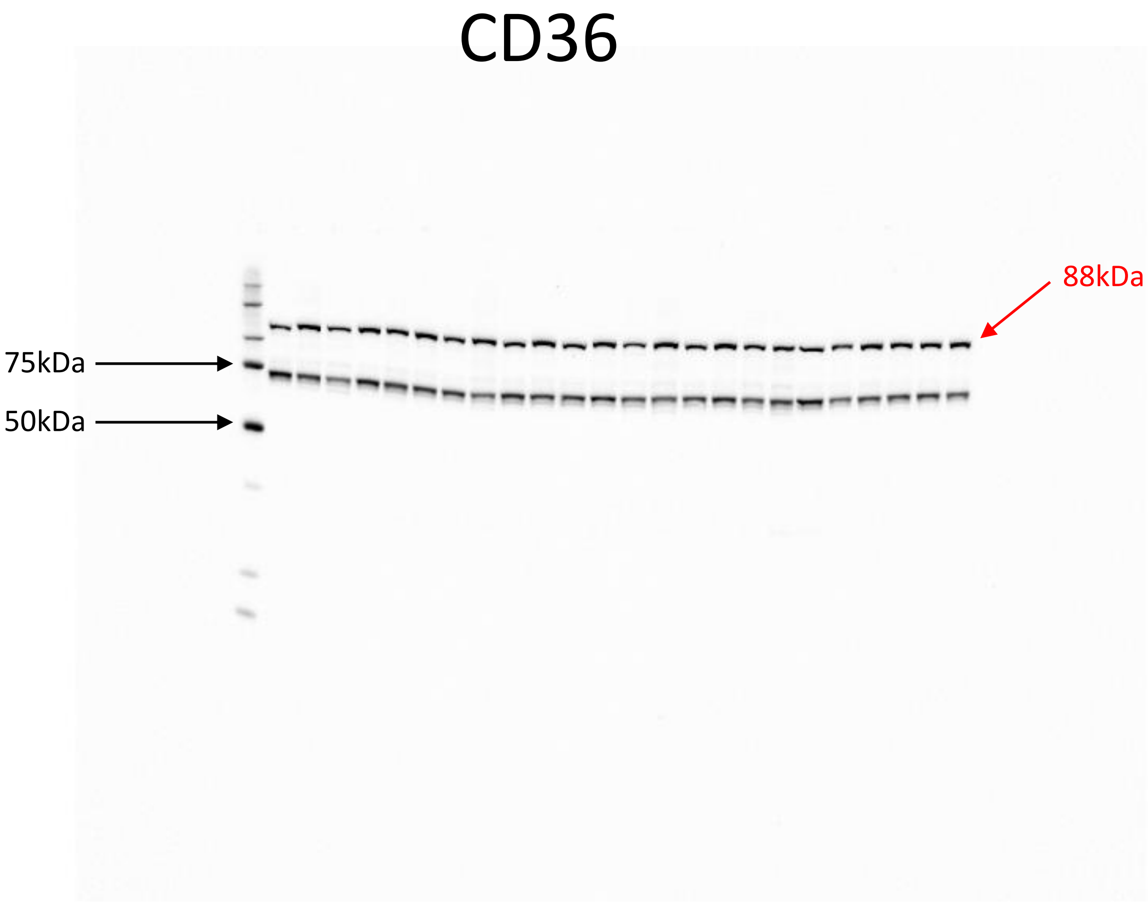
CD36
75kDa
50kDa
88kDa

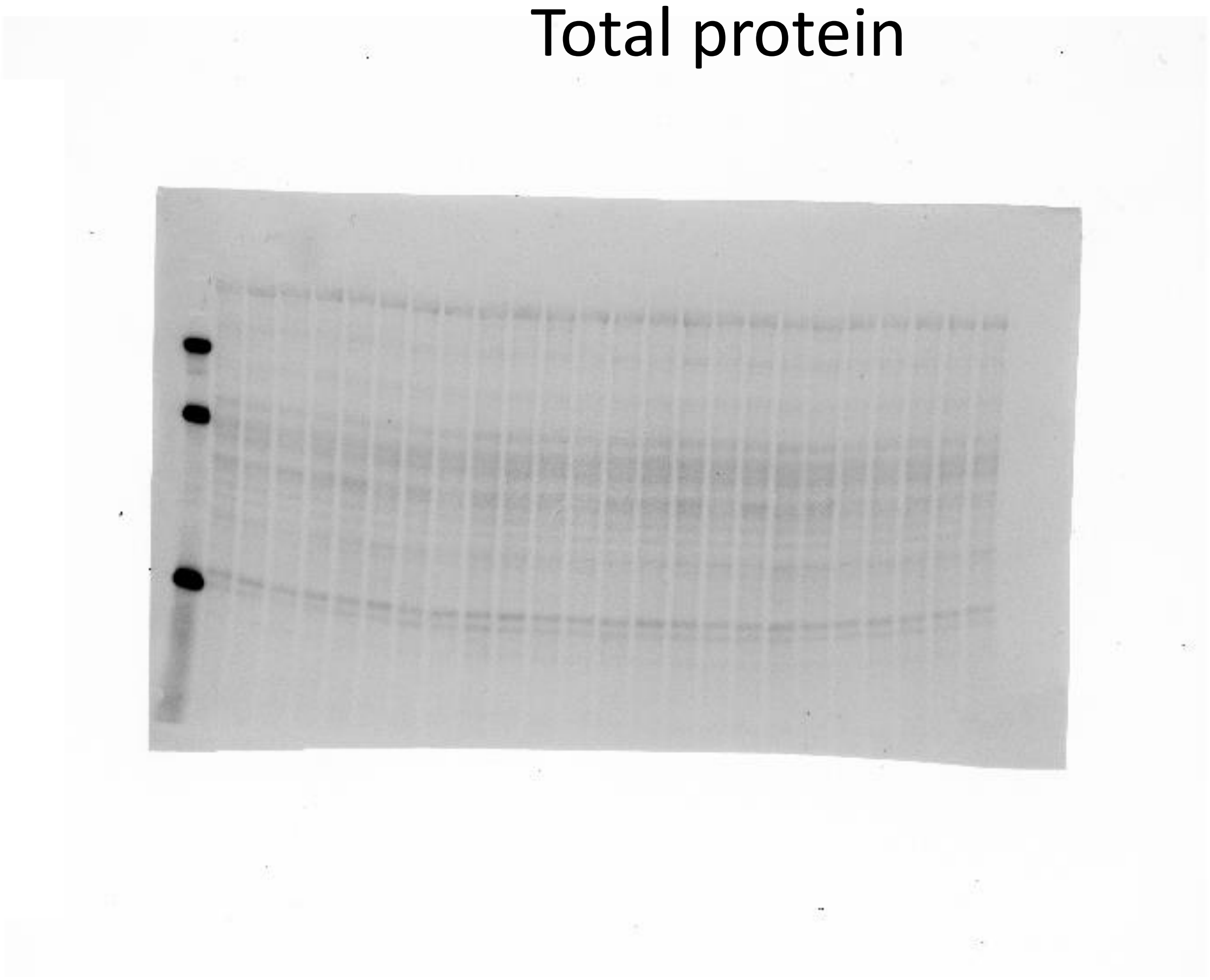
Total protein

FABPpm

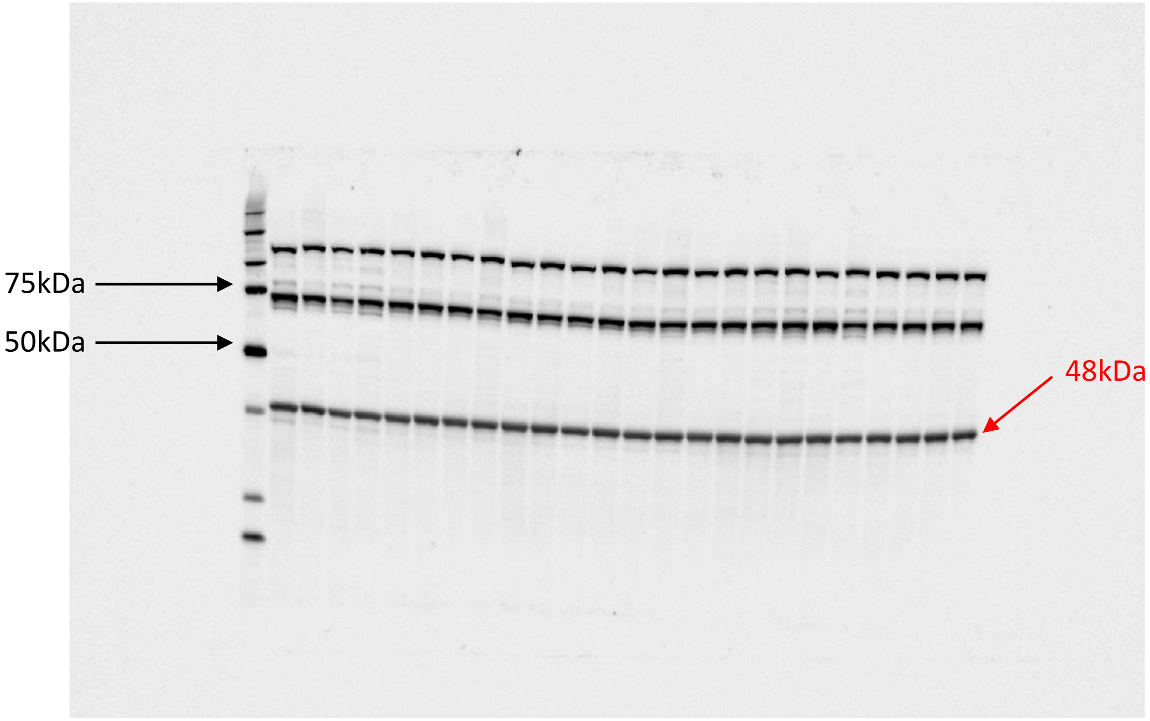

Total protein

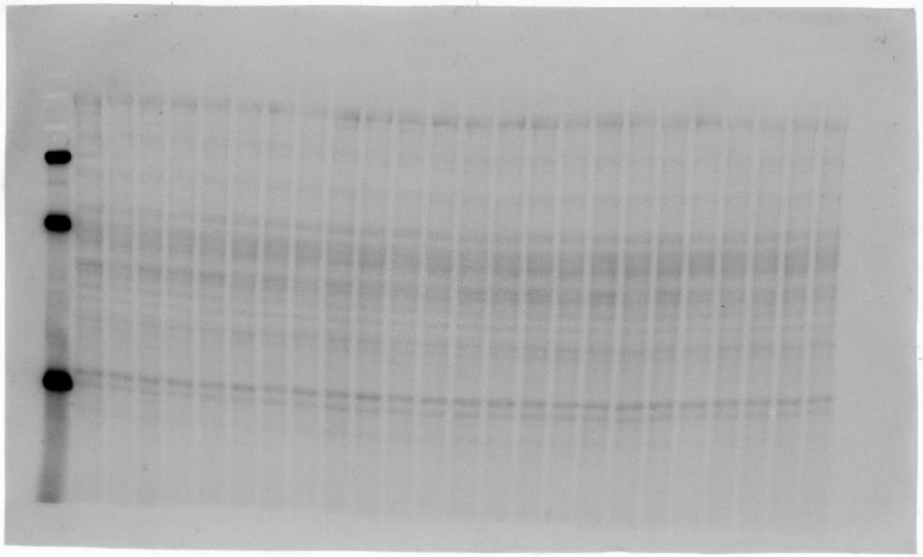

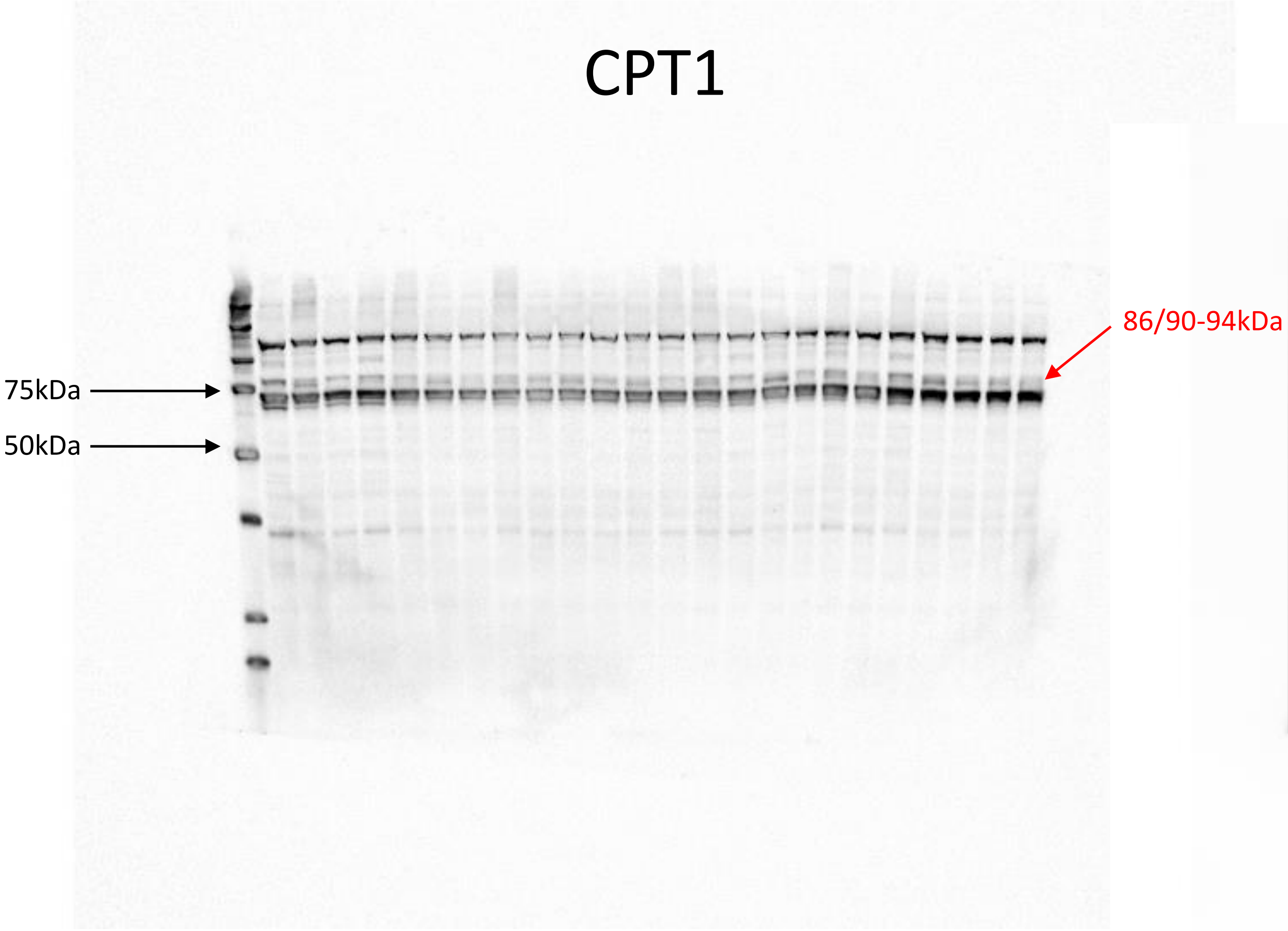
CPT1
75kDa
50kDa
86/90-94kDa

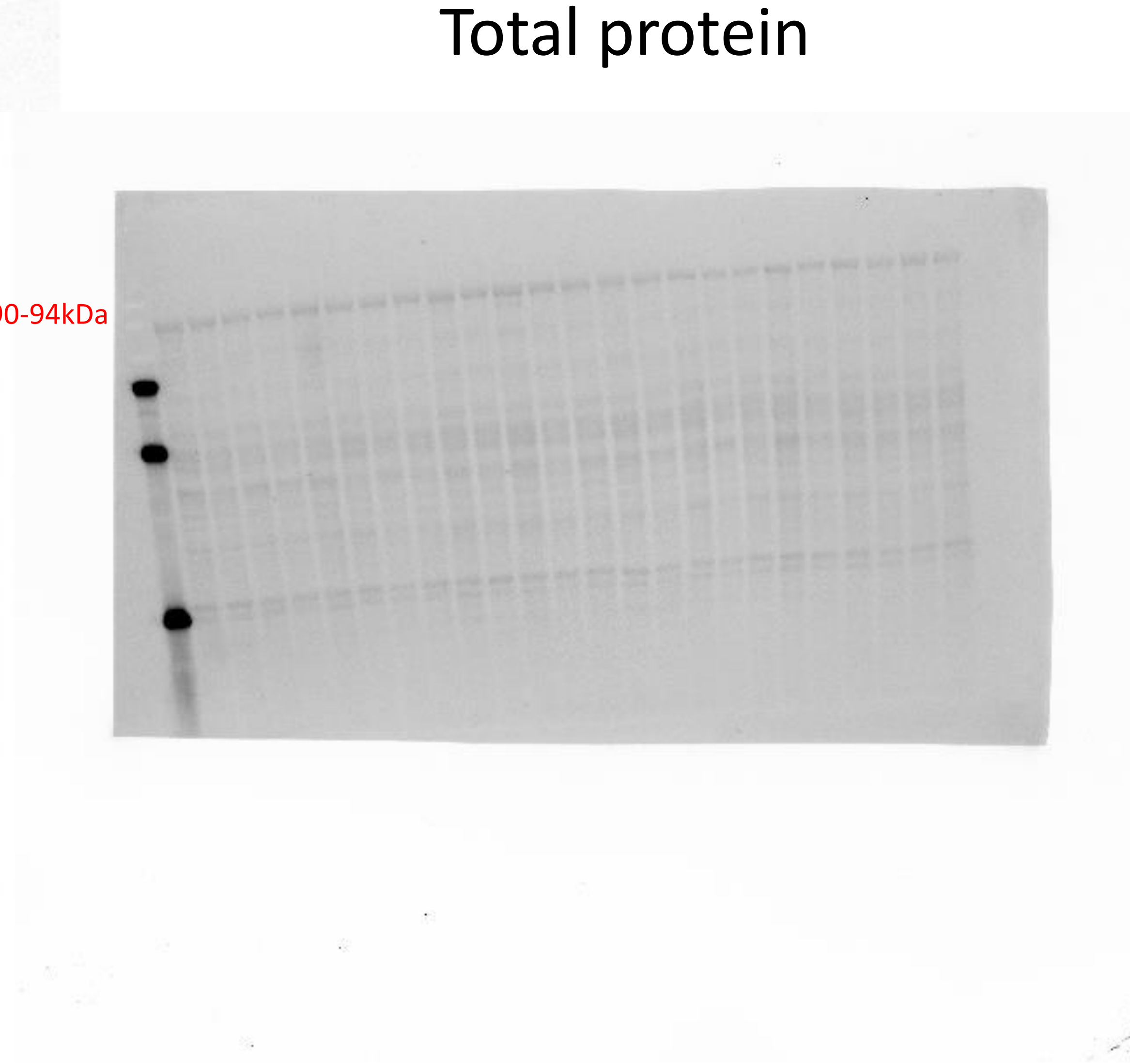
Total protein

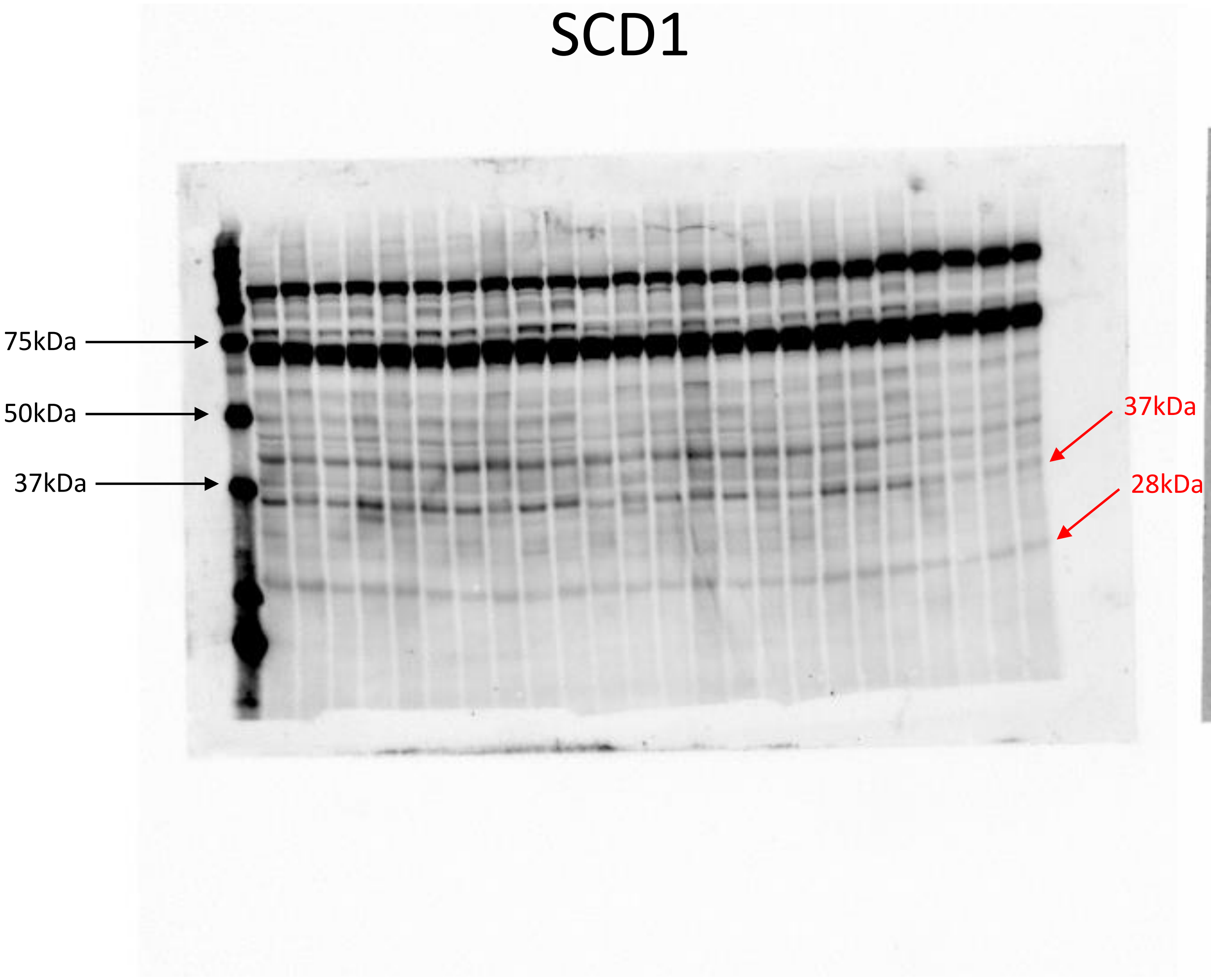
SCD1
75kDa
50kDa
37kDa
37kDa
28kDa

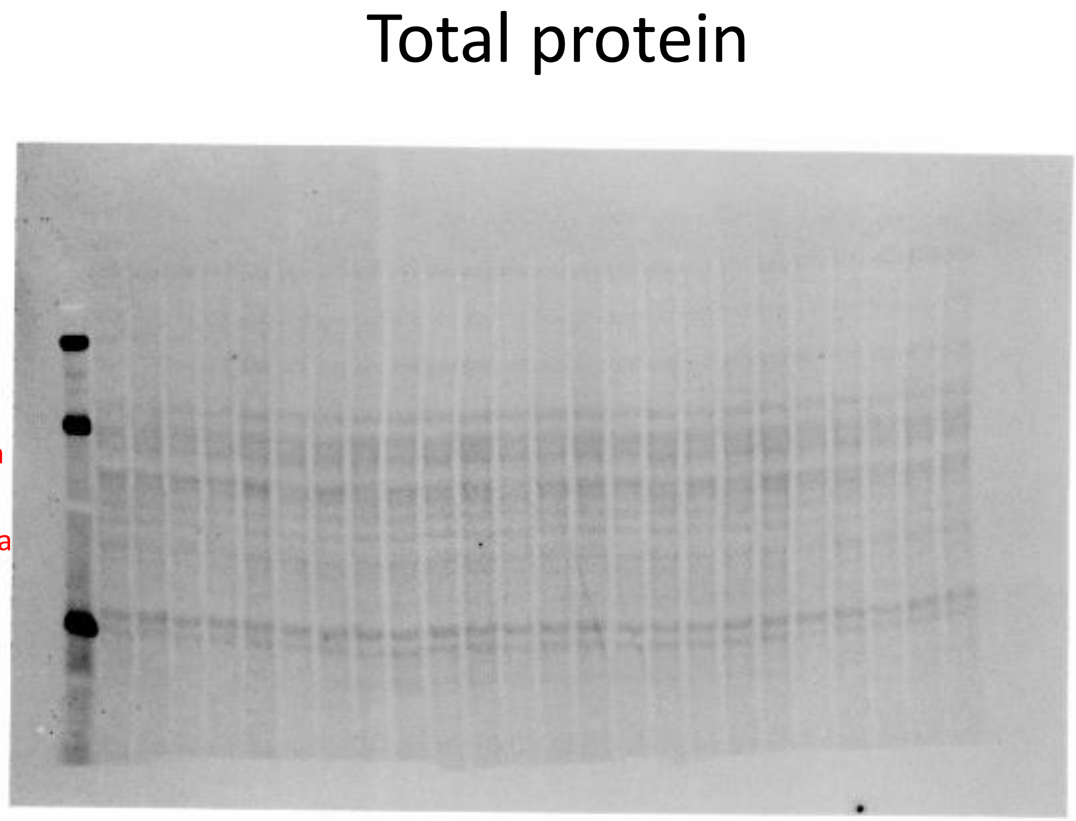
Total protein

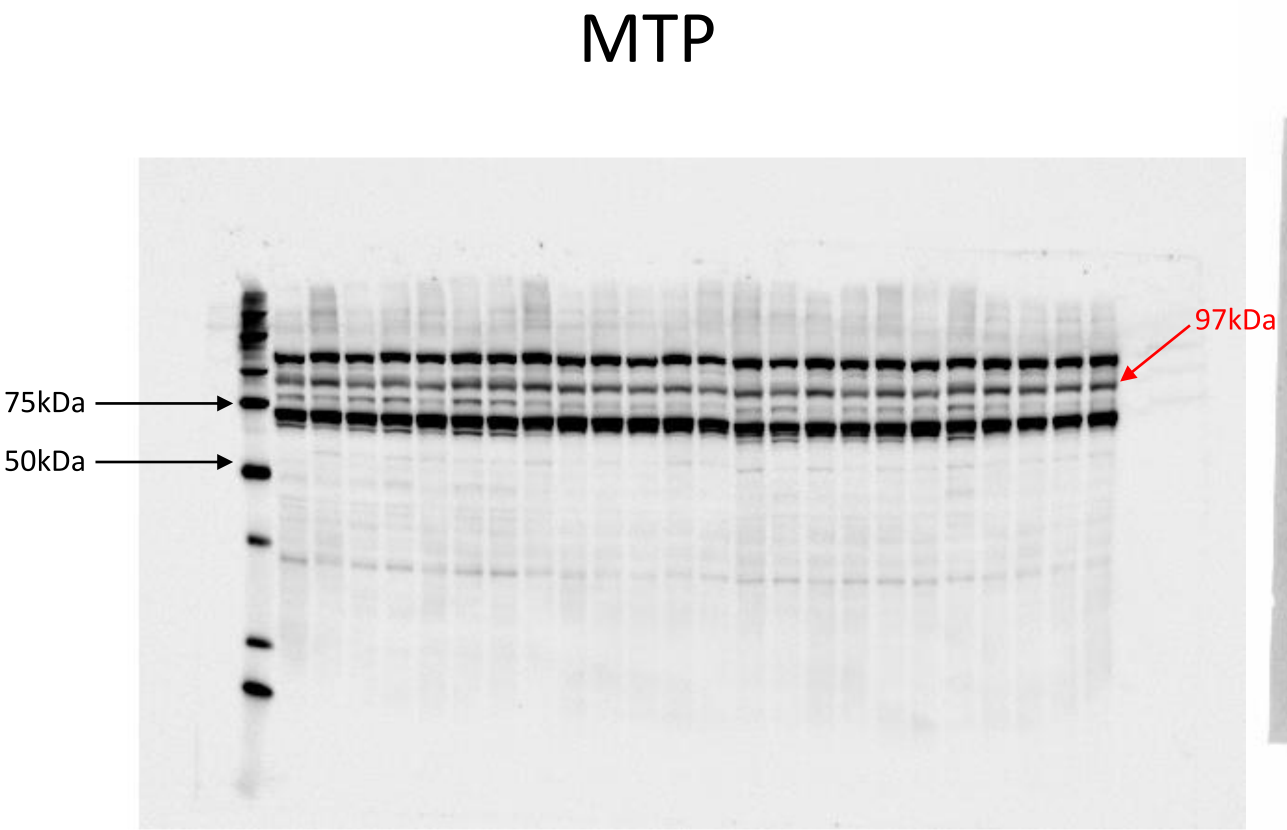

MTP
75kDa
50kDa
97kDa

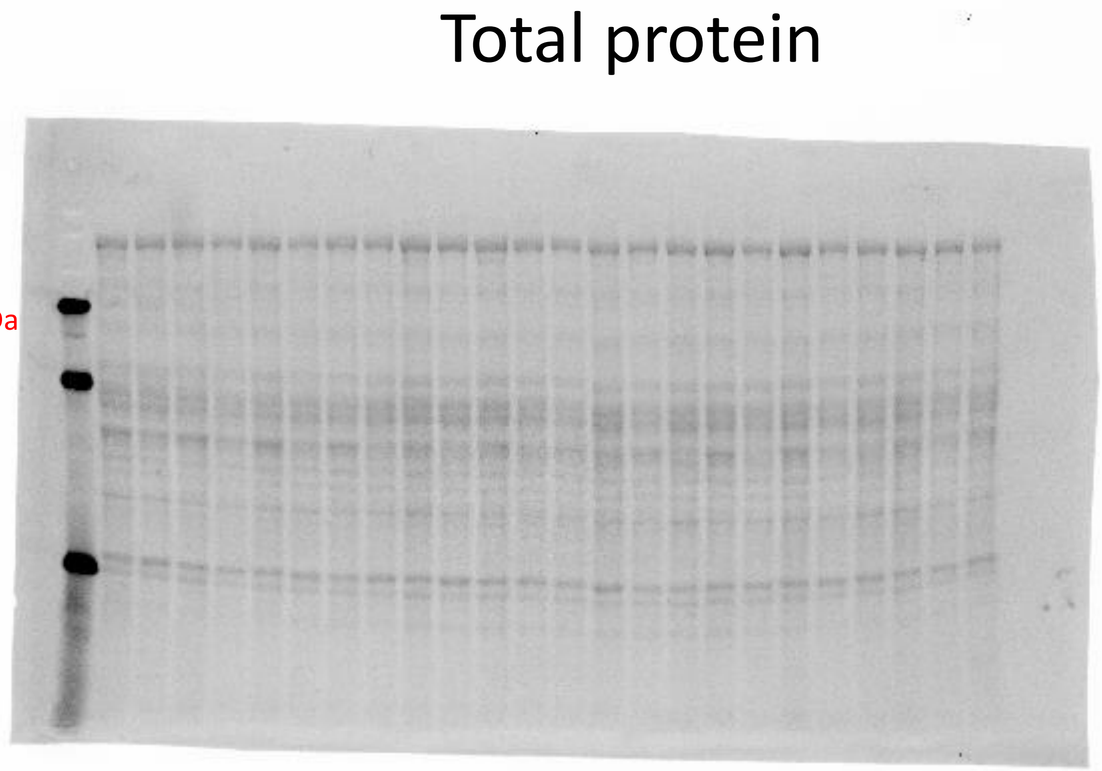

Total protein

CS

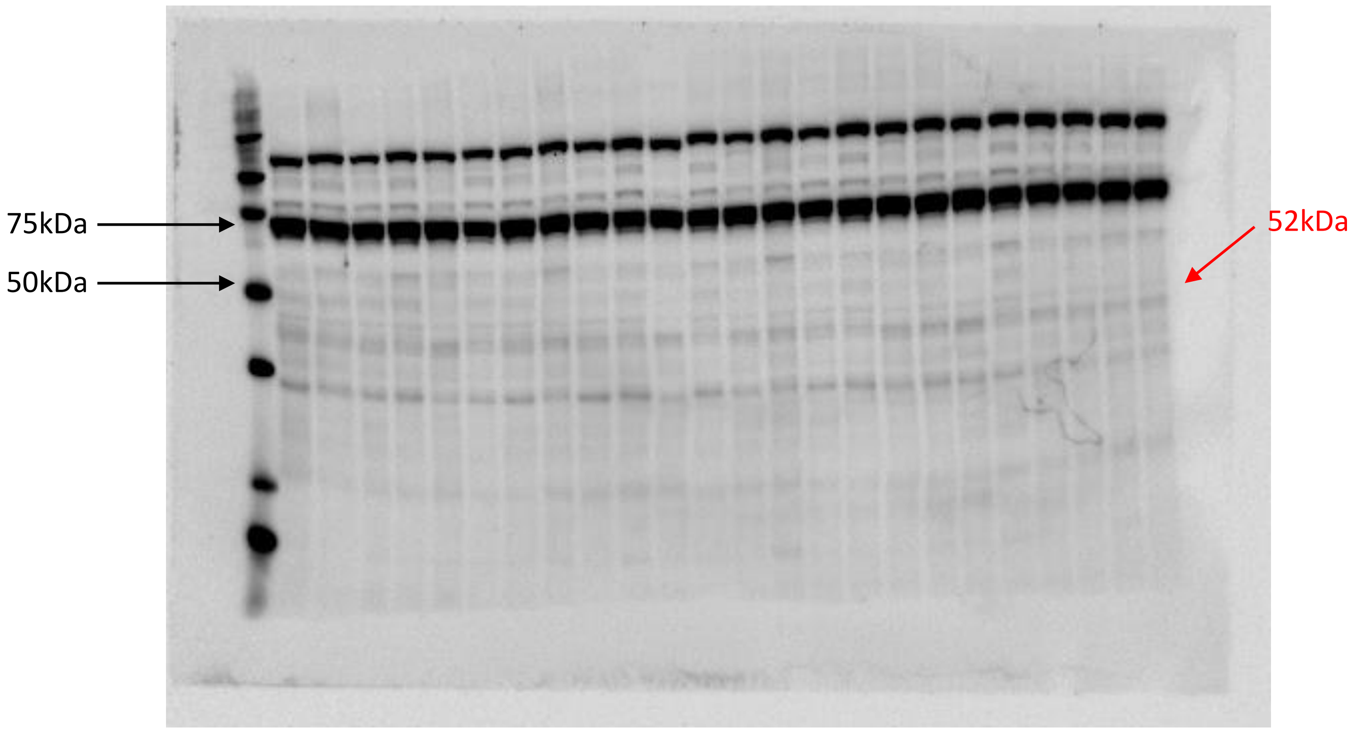

Total protein

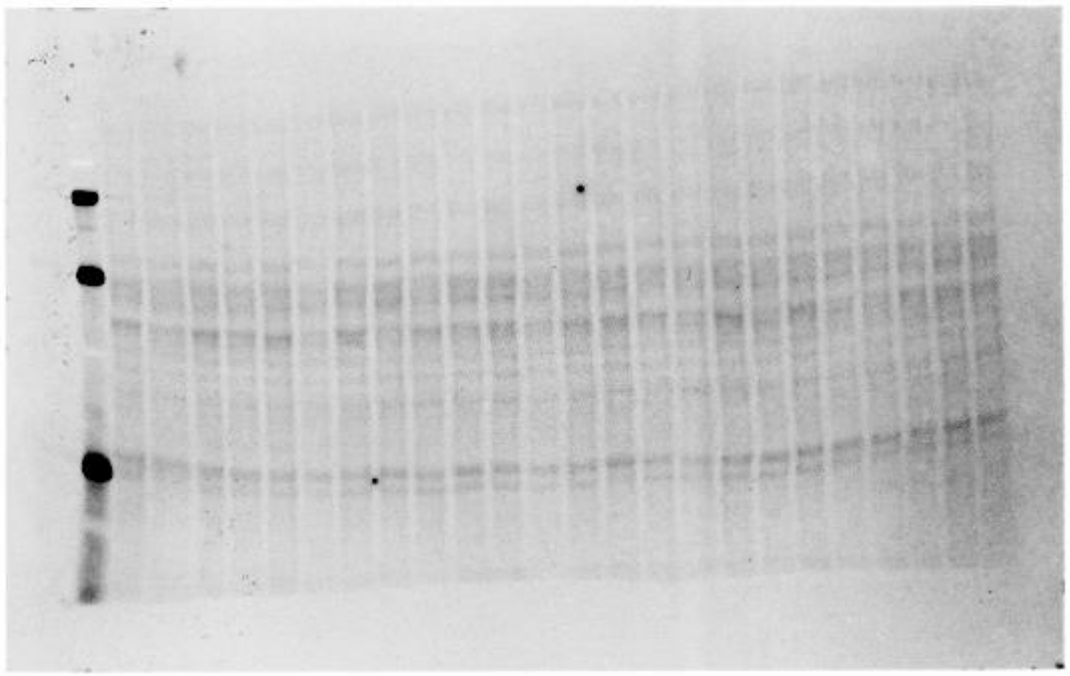

β-HAD

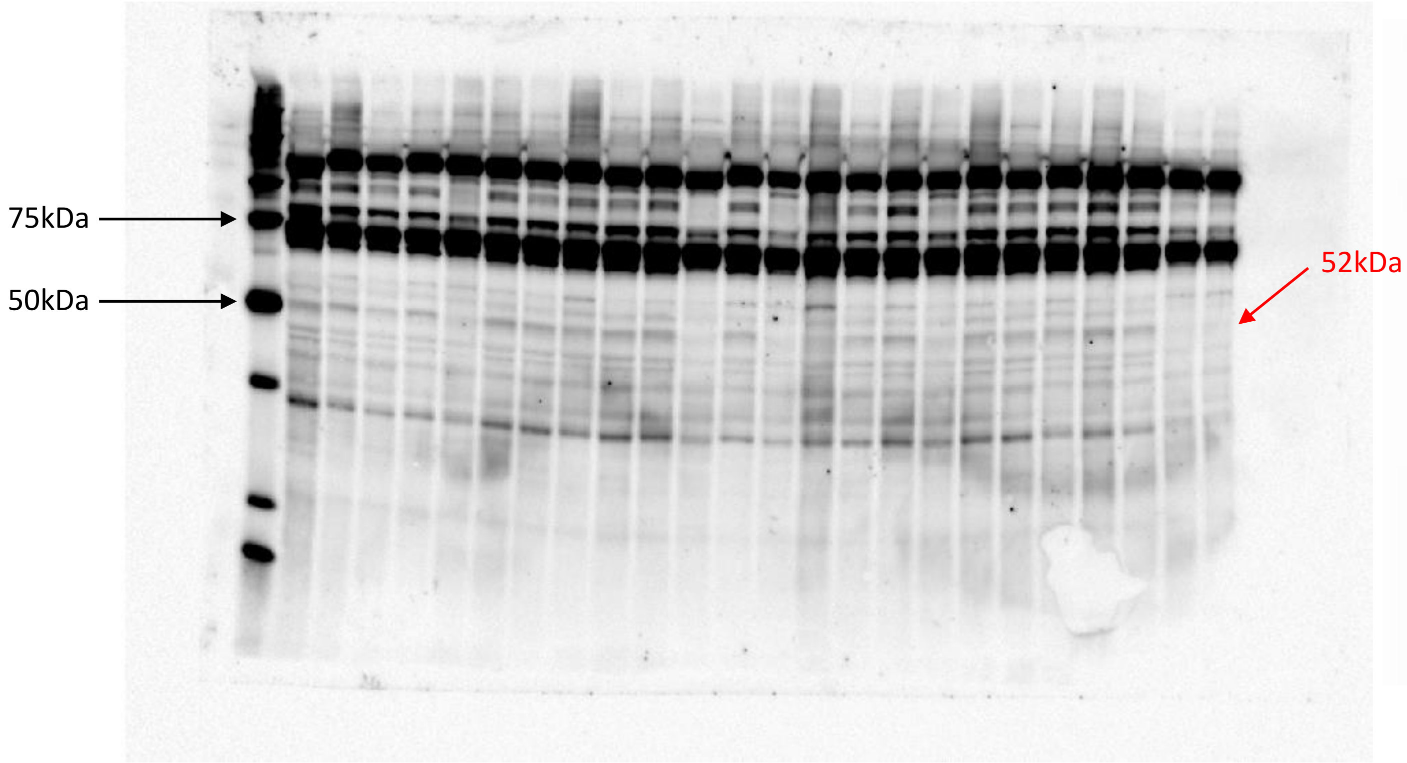

Total protein

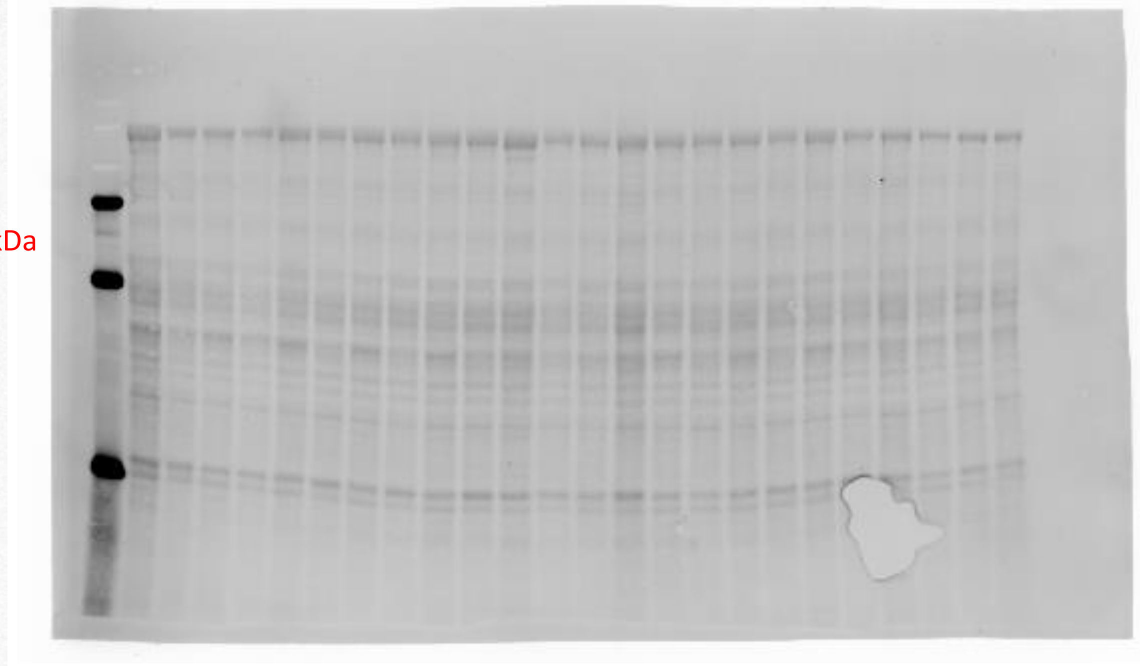

FAS

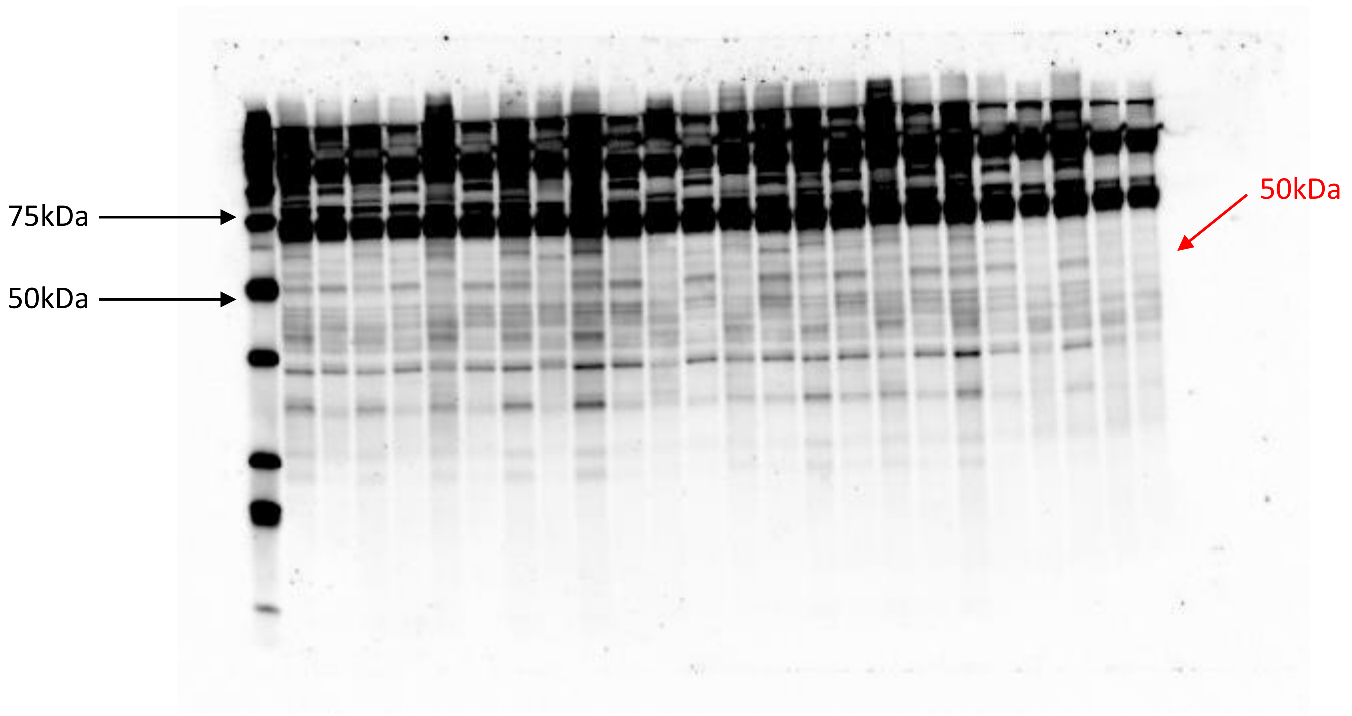

Total protein

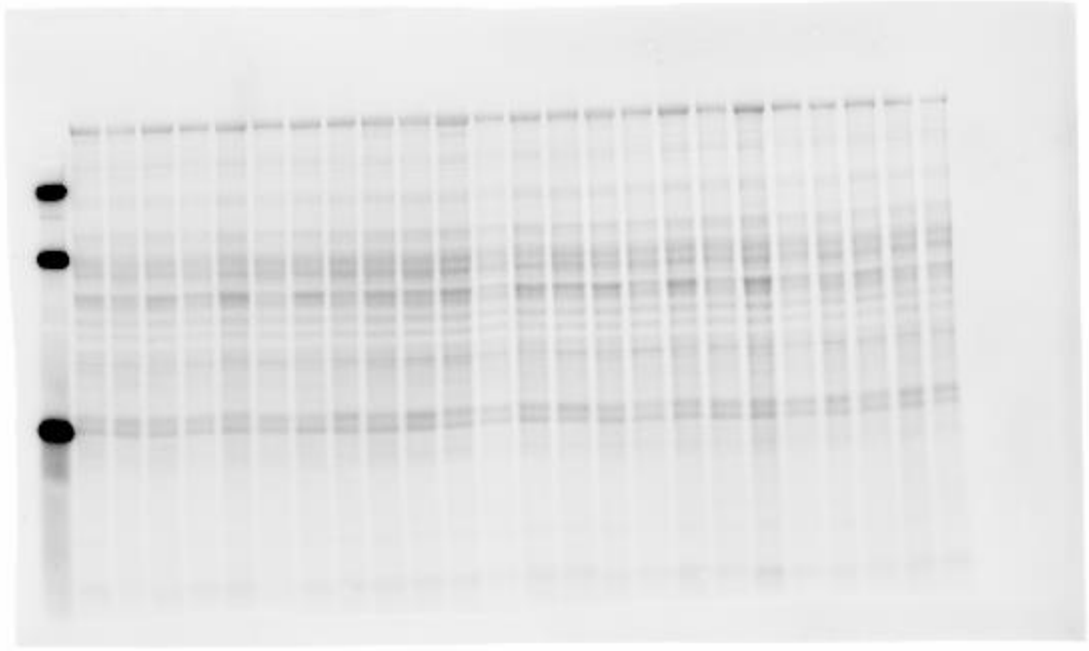

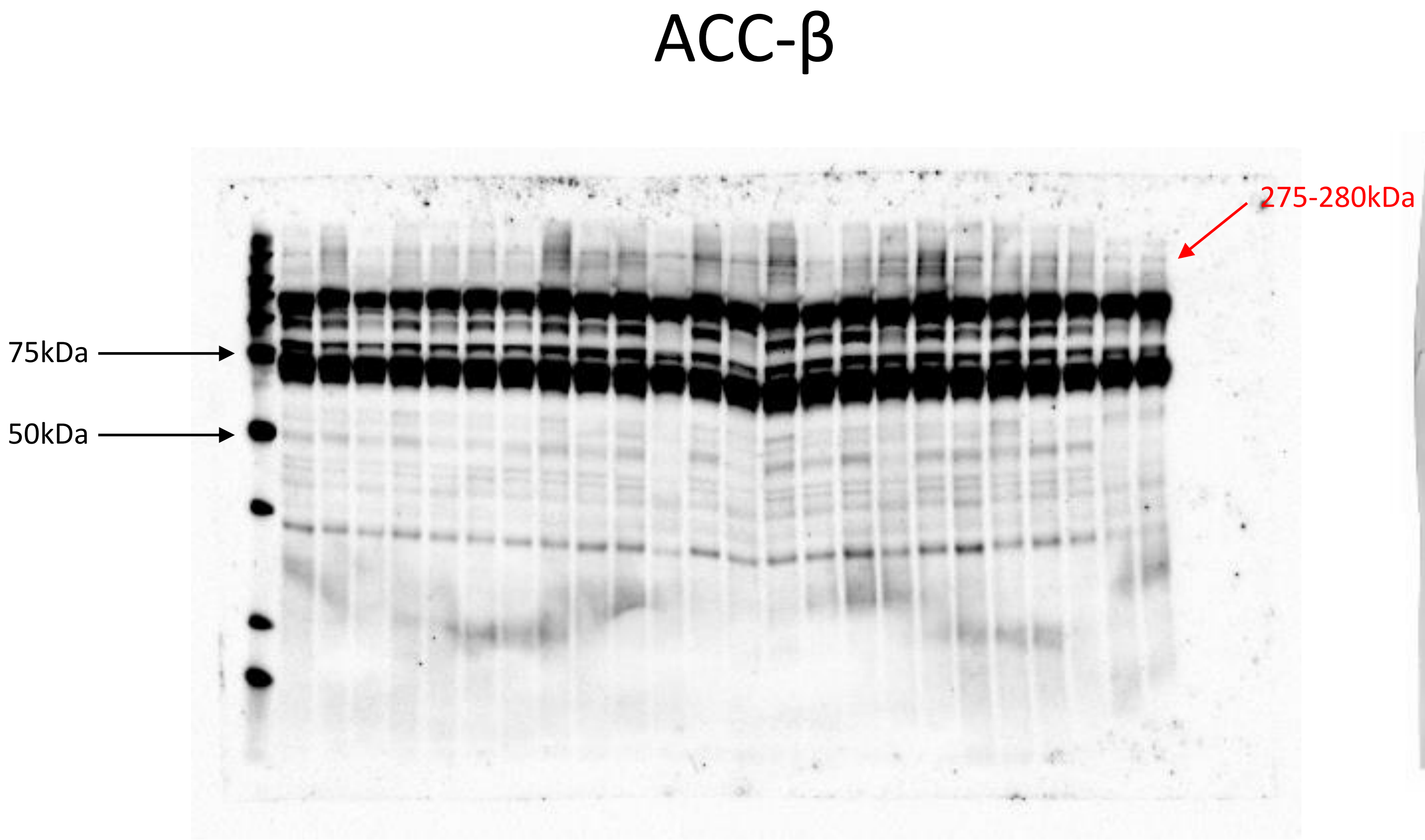
ACC-β
75kDa
50kDa
275-280kDa
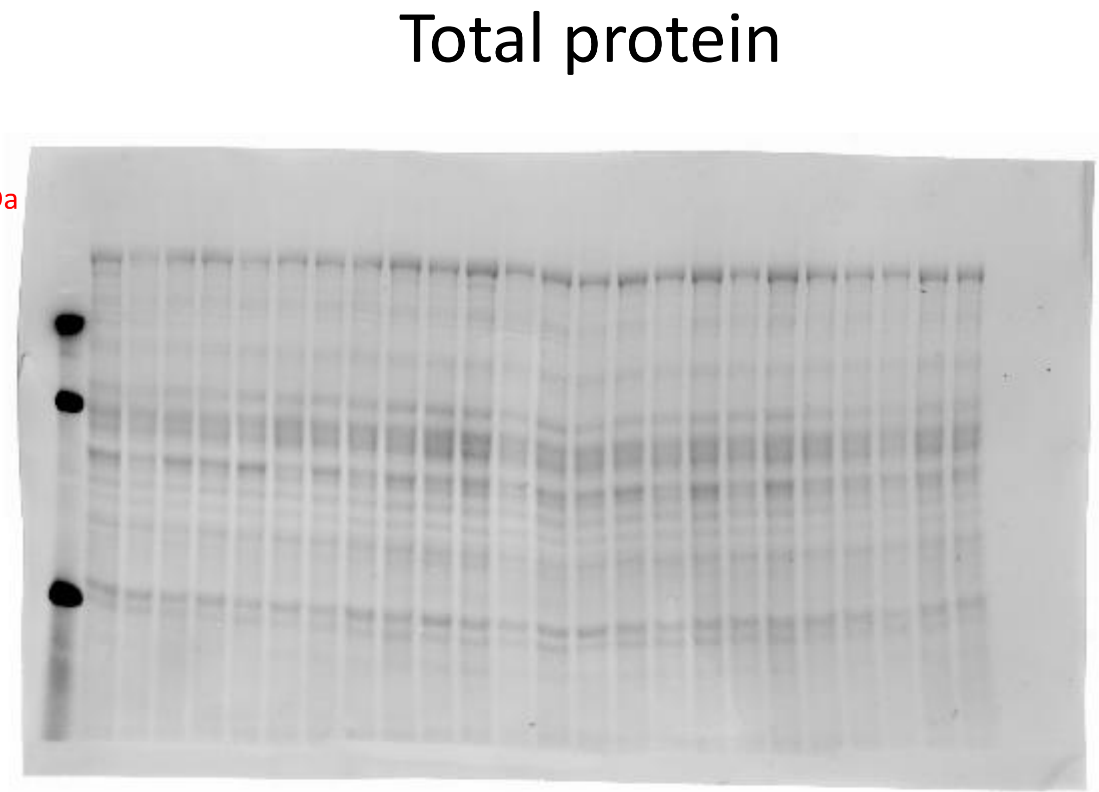
Total protein

FADS1

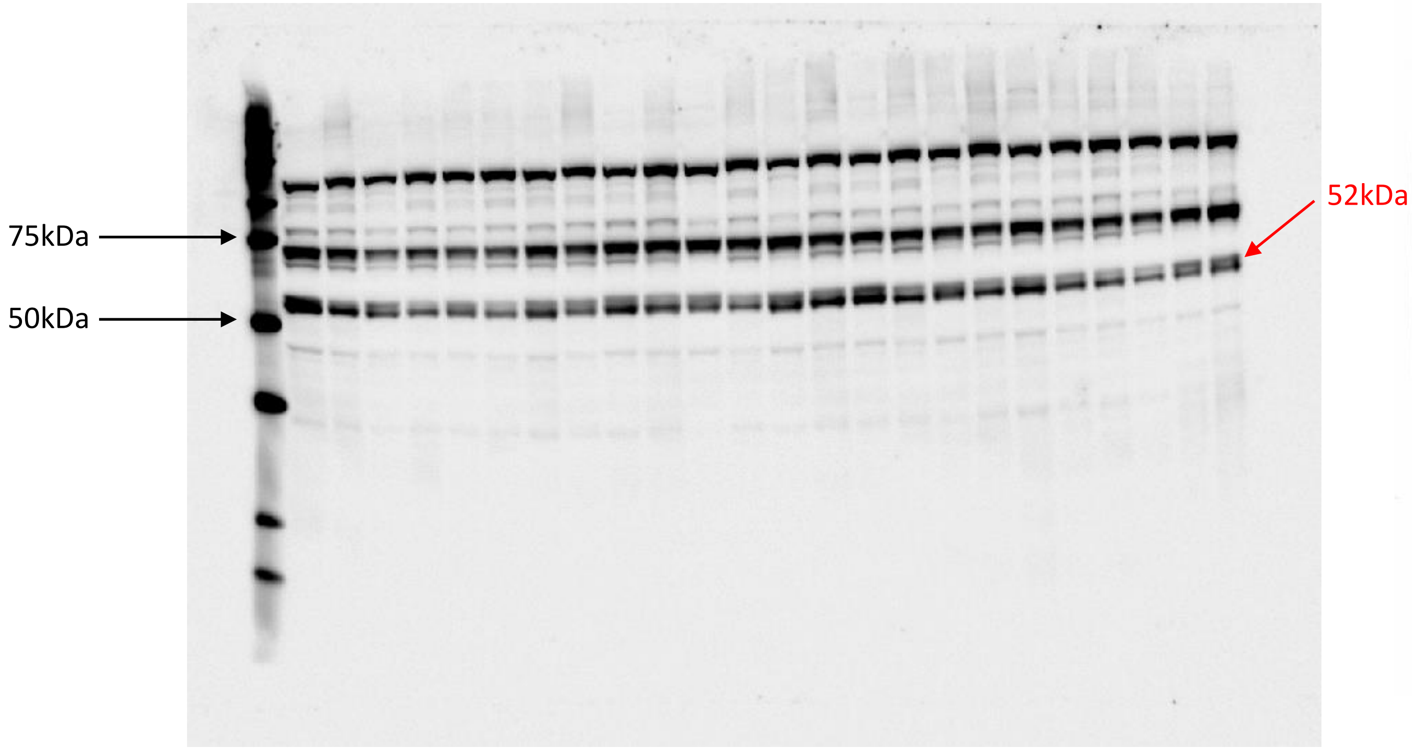

Total protein

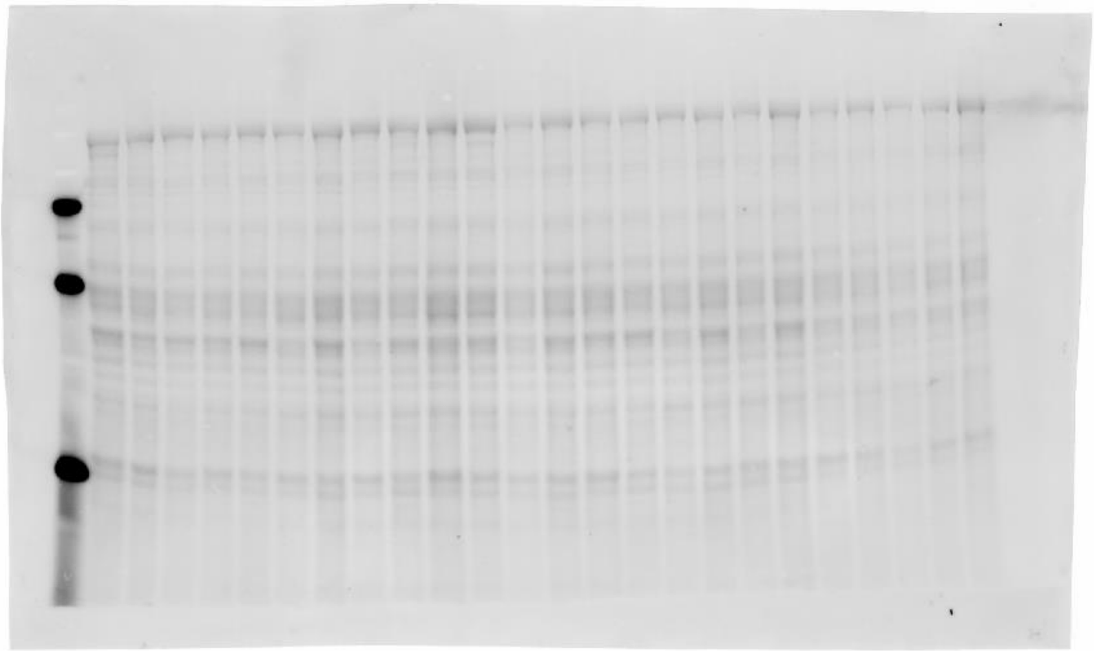

FADS2

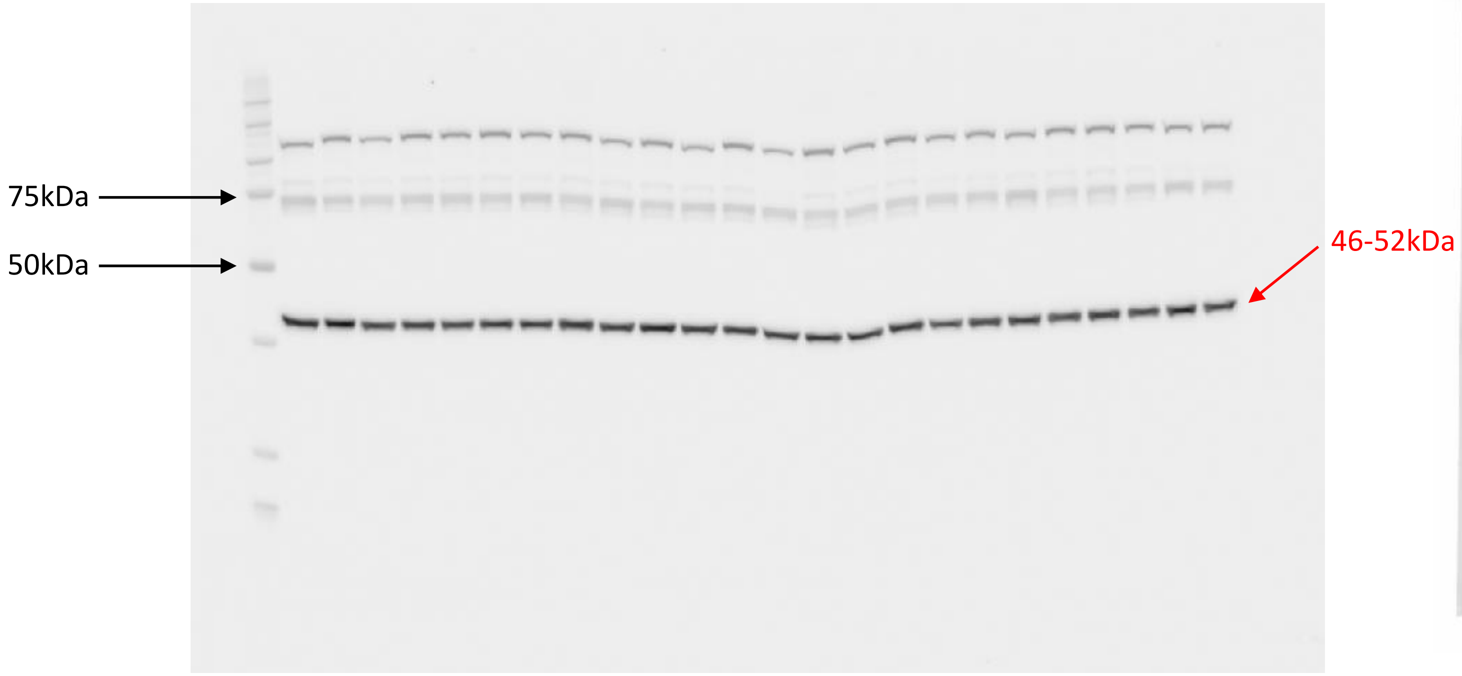

Total protein

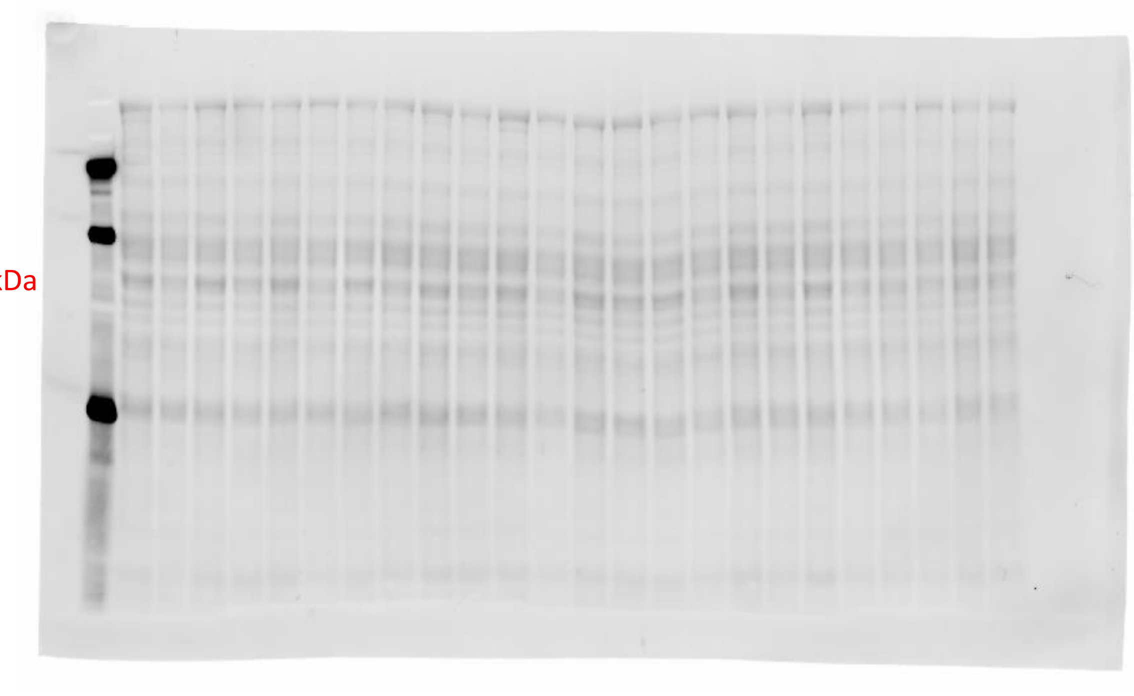

SREBP-1

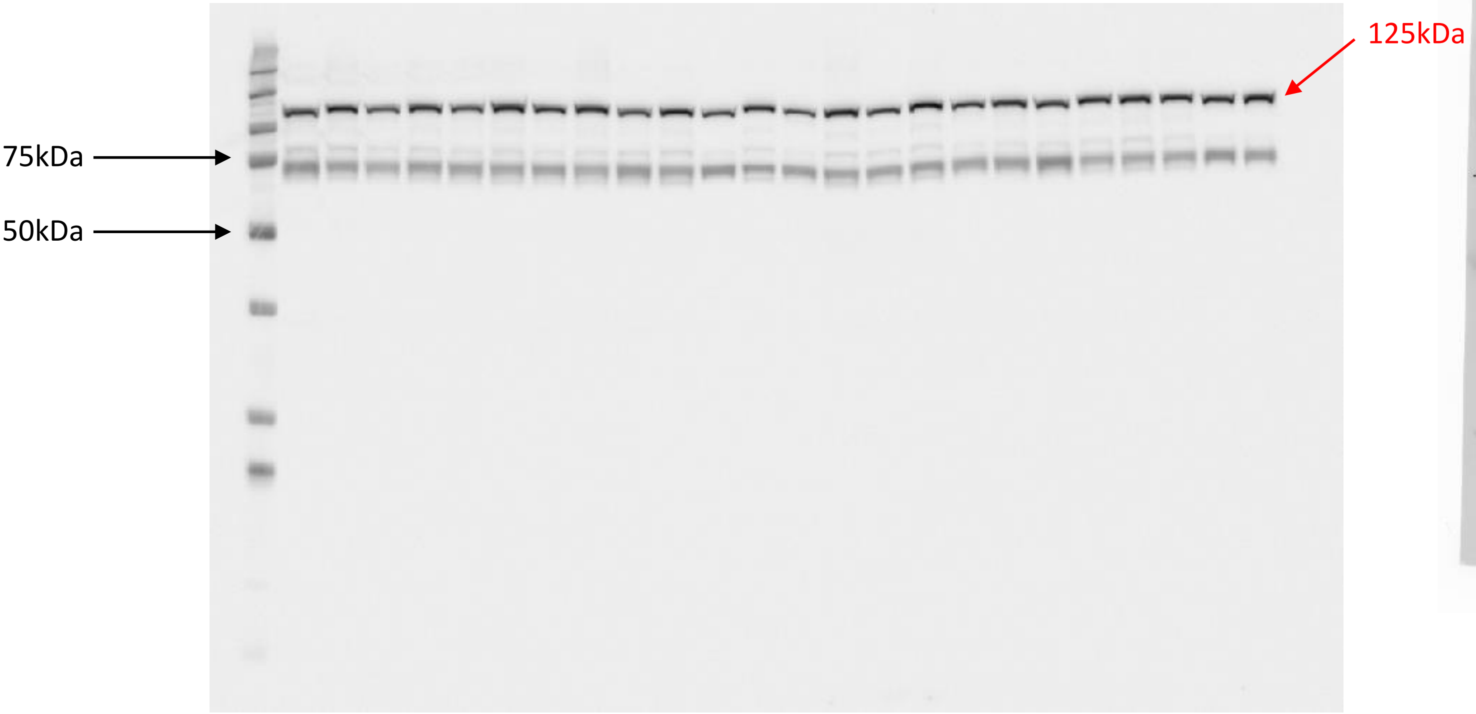

Total protein

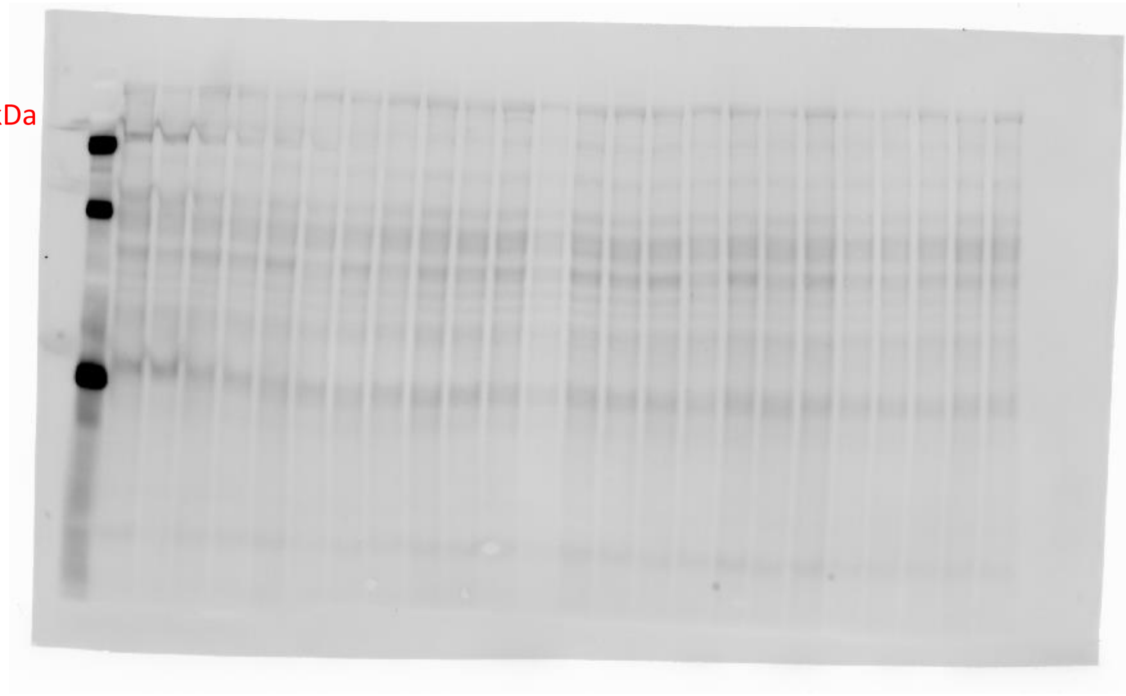

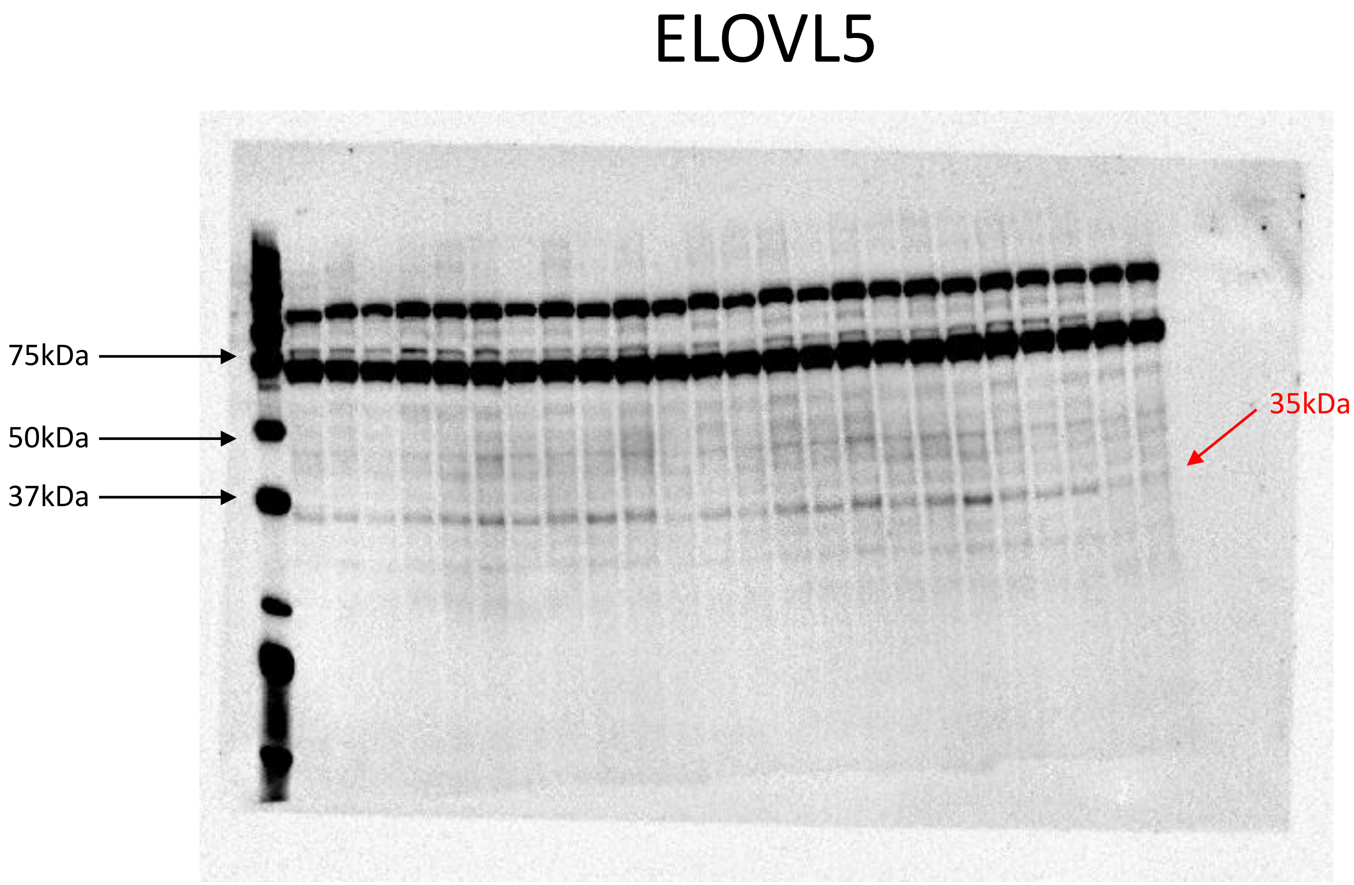
ELOVL5
75kDa
50kDa
37kDa
35kDa

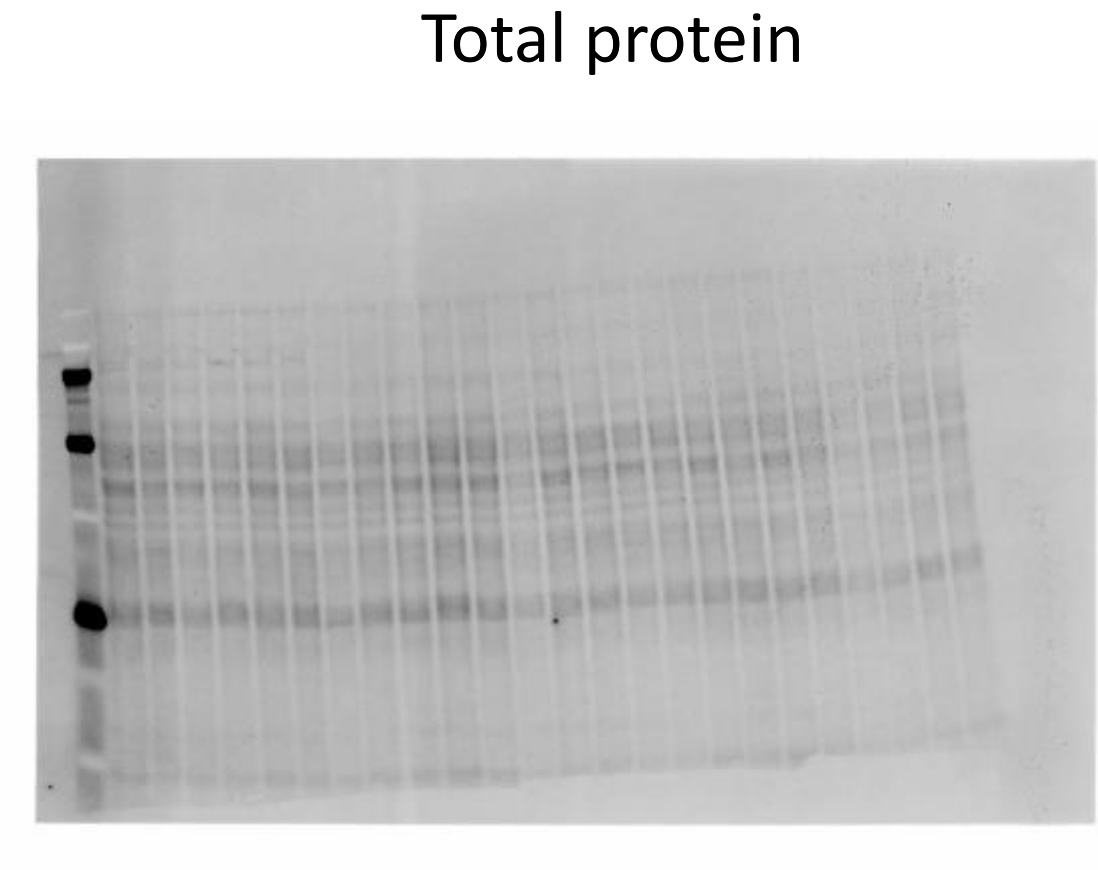
Total protein

ELOVL6

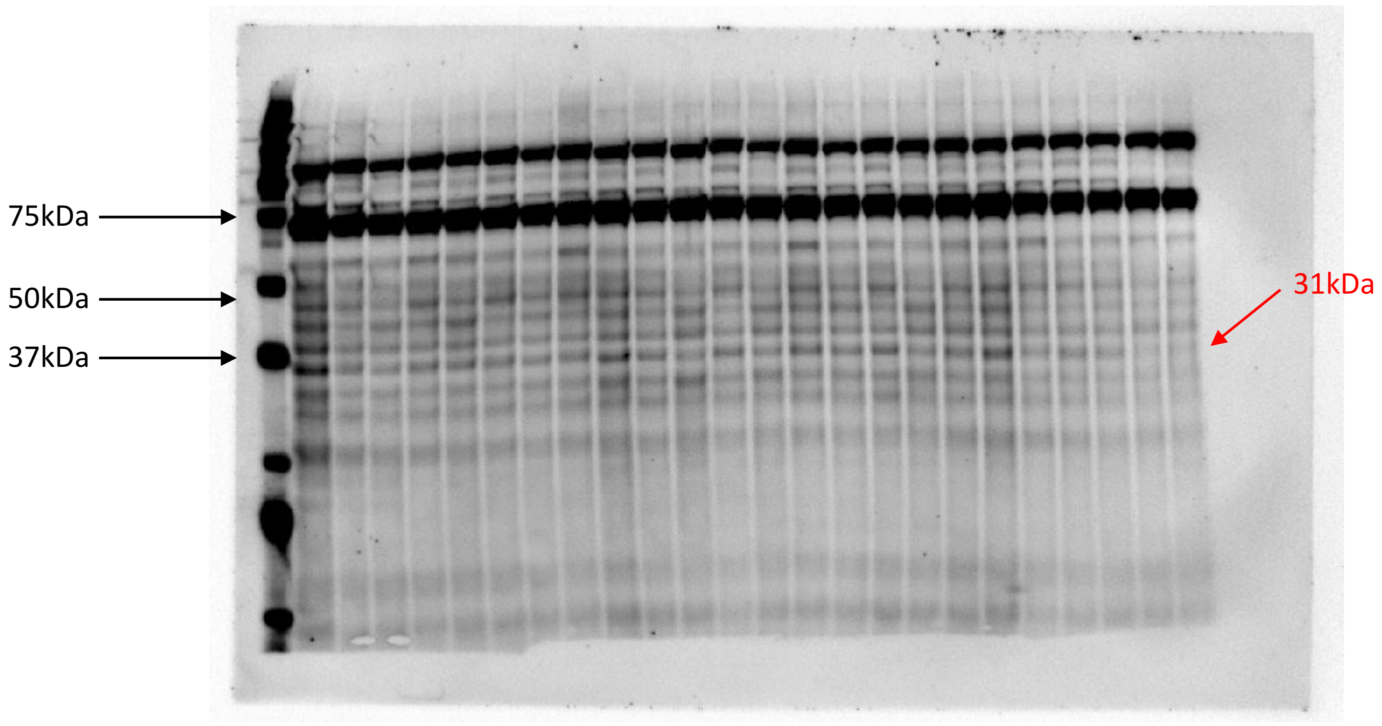

Total protein

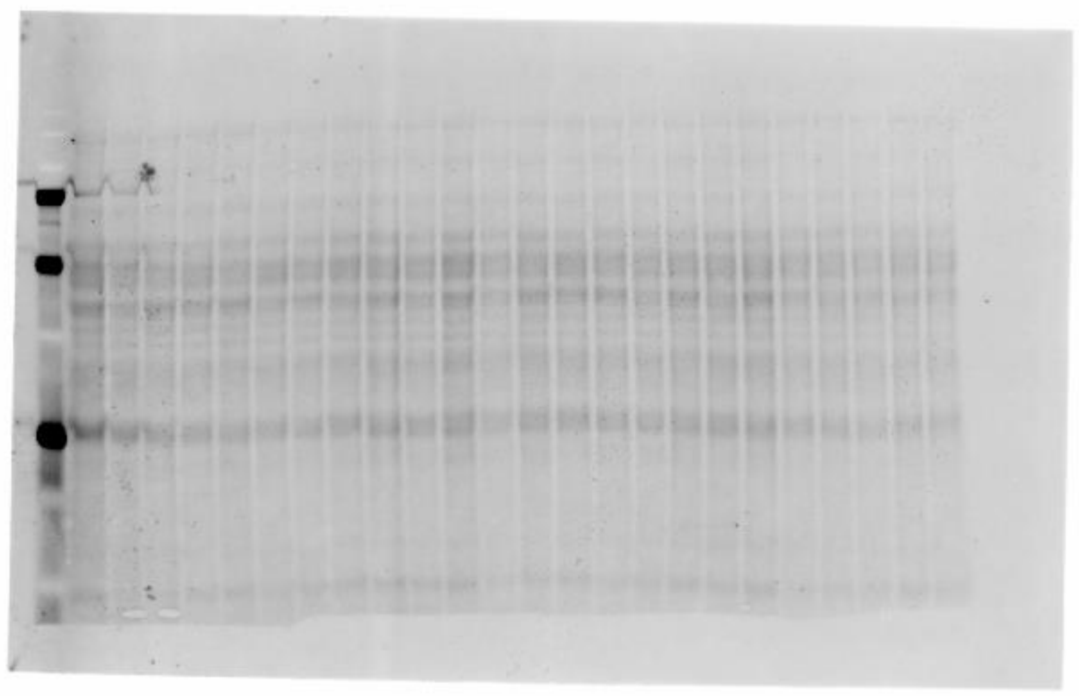

Supplement: Supplementary file 3 — Supplementary Material 3 [file 41598_2026_55583_MOESM3_ESM.pdf]
